# Population dynamics of engineered underdominance and killer-rescue gene drives in the control of disease vectors

**DOI:** 10.1371/journal.pcbi.1006059

**Published:** 2018-03-23

**Authors:** Matthew P. Edgington, Luke S. Alphey

**Affiliations:** The Pirbright Institute, Pirbright, Woking, Surrey, United Kingdom; Max-Planck-Institute for Evolutionary Biology, GERMANY

## Abstract

A number of different genetics-based vector control methods have been proposed. Two approaches currently under development in *Aedes aegypti* mosquitoes are the two-locus engineered underdominance and killer-rescue gene drive systems. Each of these is theoretically capable of increasing in frequency within a population, thus spreading associated desirable genetic traits. Thus they have gained attention for their potential to aid in the fight against various mosquito-vectored diseases. In the case of engineered underdominance, introduced transgenes are theoretically capable of persisting indefinitely (i.e. it is self-sustaining) whilst in the killer-rescue system the rescue component should initially increase in frequency (while the lethal component (killer) is common) before eventually declining (when the killer is rare) and being eliminated (i.e. it is temporally self-limiting). The population genetics of both systems have been explored using discrete generation mathematical models. The effects of various ecological factors on these two systems have also been considered using alternative modelling methodologies. Here we formulate and analyse new mathematical models combining the population dynamics and population genetics of these two classes of gene drive that incorporate ecological factors not previously studied and are simple enough to allow the effects of each to be disentangled. In particular, we focus on the potential effects that may be obtained as a result of differing ecological factors such as strengths of larval competition; numbers of breeding sites; and the relative fitness of transgenic mosquitoes compared with their wild-type counterparts. We also extend our models to consider population dynamics in two demes in order to explore the effects of dispersal between neighbouring populations on the outcome of UD and KR gene drive systems.

## Author summary

Vector-borne diseases represent a severe burden to both human and animal health worldwide. The methods currently being used to control a range of these diseases do not appear sufficient to address the issues at hand. As such, alternate methods for the control of vector-borne diseases are currently being investigated. Among the promising techniques currently being considered are a range of genetic control methods known as gene drive systems. These allow desirable genetic traits (such as a much reduced capacity for vectors to transmit viruses) to be spread through a target population; taking advantage of natural mate seeking behaviour to locate vector sub-populations that can be extremely difficult for humans to locate and reach. Here we use mathematical models (parameterised to consider mosquito populations) to demonstrate the robustness of the engineered underdominance and killer-rescue classes of gene drive to different ecological factors including birth and death rates; the number and quality of breeding sites (i.e. carrying capacity); and the strength of density-dependent competition during the larval development phase. We then go on to explore the range of potential outcomes that may result from the migration of individuals between two neighbouring populations.

## Introduction

Mosquito-borne diseases represent one of the most severe public health burdens worldwide. For example, dengue viruses that are primarily transmitted by *Aedes aegypti* mosquitoes have rapidly increased in prevalence in recent years [1]. One recent study estimated that around 3.9 billion people, in over 100 countries, live in regions ‘at risk’ for dengue infections [2], with ∼390 million dengue infections per year [3] of which perhaps 50-100 million cases are symptomatic [4]. Of these cases ∼3.9 million are classified as severe and 9,000 are fatal [4]. The methods currently used to control dengue do not appear sufficient to eliminate the problem and this is exacerbated by the lack of drug treatments presently available [5]. A first dengue vaccine has recently been licensed, however it is only recommended for use in individuals over nine years of age that live in areas of high dengue burden [6, 7]. Thus, while it may prove a useful tool in some situations, it appears unlikely to be sufficient for eliminating the threat of dengue. As such, a number of additional methods for the control of dengue and other vector-borne diseases are currently being investigated.

Genetic control methods are one such alternative and, with advances in tools available to molecular biologists, have become a realistic prospect in recent years. In particular, a range of gene drive systems have been proposed that could, in theory, be used to spread desirable genetic traits through a mosquito population [10]. Each of these systems would be implemented by introducing individuals carrying the drive system into the wild where they mate with existing mosquito populations. This potentially gives them an edge over traditional control methods since they exploit the natural behaviour of mosquitoes to seek mates and find breeding/resting sites which can be extremely difficult for humans to locate and reach. These genetic systems can be classified in a number of ways that include their intended effect, persistence and invasiveness, each of which affect how they may be viewed both by the public and regulators [10]. Two such classes of gene drive that are currently under development in our research group for *Aedes aegypti* mosquitoes are two-locus engineered underdominance (UD) and killer-rescue (KR). These have both attracted attention due to their potential to mitigate a number of key regulatory concerns.

**Fig 1:**
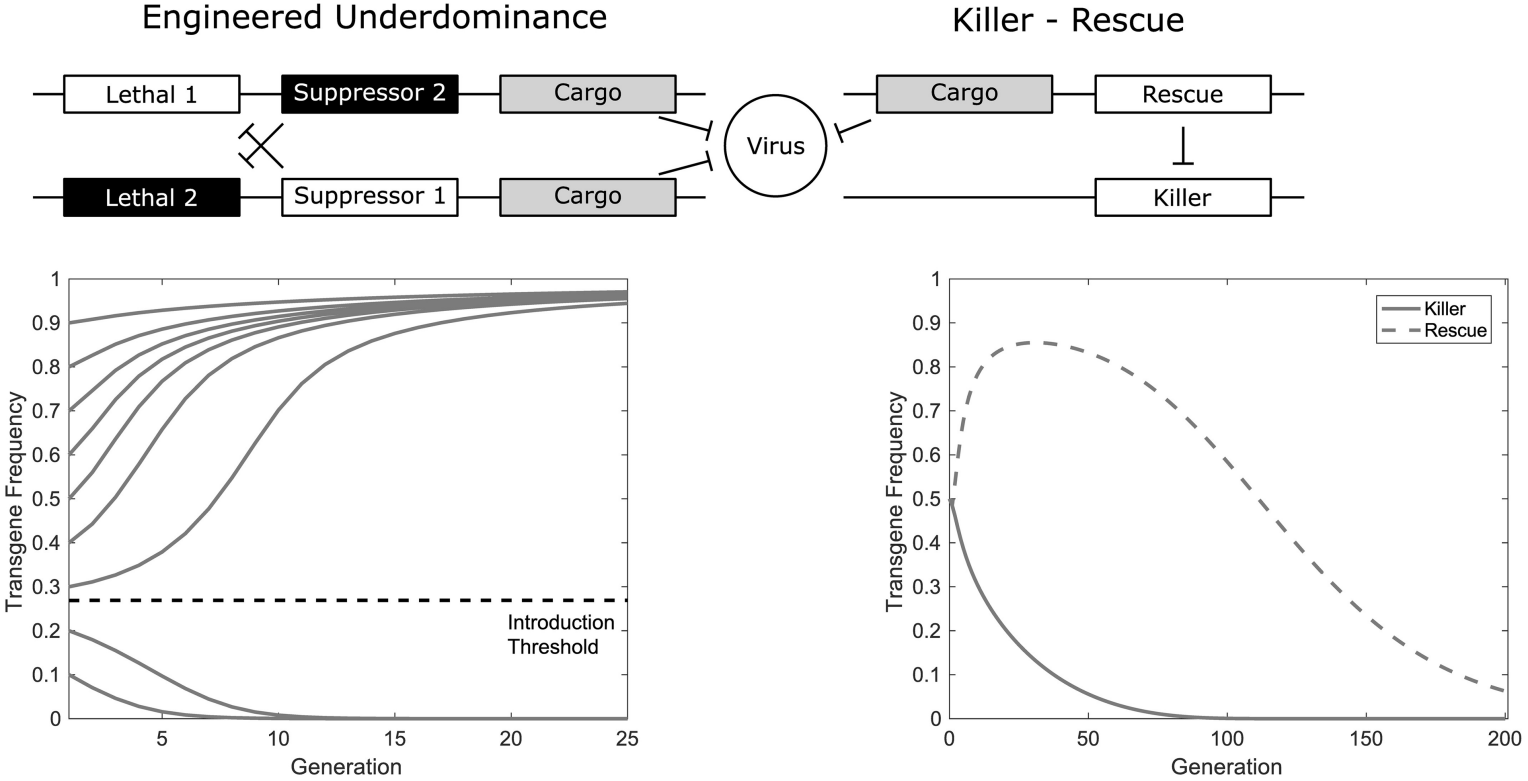
Schematic diagrams of the two-locus engineered underdominance (top left) and the killer-rescue (top right) gene drive systems. Each system comprises of two independently inherited transgenic constructs carrying different configurations of lethal (killer), suppressor (rescue) and cargo (refractory) genes. In the case of engineered underdominance each construct carries a lethal gene, the cargo (refractory) gene and a suppressor of the lethal at the other locus. This differs from the killer-rescue system in which one construct carries a killer gene while the other carries the suppressor and a cargo (refractory) gene. The engineered underdominance system considered here can be thought of as two orthogonal killer-rescue systems split across two transgenic constructs. The engineered underdominance system has been shown to be threshold dependent (bottom left) and persist in time (i.e. it is self-sustaining) whereas the killer-rescue (bottom right) system initially increases in frequency before being eliminated from the population (i.e. it is self-limiting, even in absence of genetic changes such as resistant alleles or mutation of system components).

Underdominance is a phenomenon most commonly thought of in the context of two or more alleles at a single genetic locus. It refers to the scenario whereby individuals heterozygous at a given genetic locus are less fit than either of the homozygote states and is the inverse of the better known hybrid vigour. UD systems, as proposed by Davis et al. [8], seek to attain a similar effect through a reduction in the fitness of hybrids between parental strains. This is achieved via the introduction of two independently inherited transgenic constructs inserted at unlinked loci (Fig 1). Each of these transgenic constructs carries a lethal genetic element, a suppressor for the lethal at the other locus and a “cargo” gene conferring a desirable phenotype. The requirement for lethal effectors within UD systems leads to a number of challenges for design and construction of such systems. It is theoretically possible to engineer these systems sequentially. However, their development and testing will be much simpler if lethal genes are either conditionally lethal, repressible or incompletely lethal. Within this study we restrict our attention to cargo genes that render individuals refractory to one or more viruses (i.e. a reduced vector competence trait), significantly reducing their capacity for infecting humans. Further, this trait is assumed to be dominant meaning it will be fully effective in a single copy. Such refractory genes have already been developed, for example those of Franz and colleagues [11, 12], with further examples likely in development. Under such a system [8], individuals (other than wild-type) that do not inherit at least one copy of each transgenic construct are non-viable because they carry one of the lethal genes but lack the relevant suppressor. This creates a selection pressure for individuals to carry both constructs or neither. Under certain conditions this can theoretically allow the transgenic constructs, and crucially, the cargo (refractory) gene(s) to spread toward fixation within a population if they are introduced at a sufficiently high frequency. Due to the ongoing selection pressure for individuals to carry both transgenic constructs, it is expected that, in absence of resistance or mutation, the introduced transgenes will persist indefinitely. Thus, it is anticipated that in such situations the prevalence of infections within a targeted area could be significantly reduced, or even eliminated.

The UD system is essentially composed of two orthogonal KR systems [9], and a key developmental milestone will be the construction and testing of one/both KR system(s). Hence, we consider here the behaviour of this class of gene drive system also. In this case one lethal (killer) and one suppressor (rescue) gene are independently inherited and inserted at unlinked loci (Fig 1). As long as it is common within the population, the deleterious effect of the lethal transgene creates a selective pressure for individuals to also carry copies of the suppressor gene. This causes the suppressor (rescue) construct to increase in frequency within the population. However, this selective pressure is diminished as the lethal becomes more rare (due to fitness costs). Thus the suppressor too begins to reduce in frequency (assuming it confers some fitness cost). In theory this eventually leads to elimination of both transgenic constructs and thus a return to the pre-release state. Such systems are interesting for the control of dengue vectors since they may reduce the number of mosquitoes capable of transmitting viruses sufficiently to disrupt disease transmission. If so, the pathogen could be eliminated without removing the mosquito species from its ecological niche.

The power of such systems to alter natural populations has led to some concerns about ways in which they should be regulated to ensure they are used safely [13, 14]. Thus it is important to consider how such systems may be viewed in terms of a number of key issues. Questions considered important in this context include the following. Will a gene drive spread into regions that did not authorise the use of genetically modified mosquitoes? If so, what effects will they have in the non-target area? Can the gene drive be reversed/recalled in the event of unanticipated/undesirable effects? These questions will be addressed in the work presented here.

Previous modelling work has demonstrated that—in absence of resistance or mutation—UD systems should persist indefinitely provided that they are introduced above some threshold transgene frequency [8, 15]. Below this threshold the transgene will simply be eliminated from the population due to both the associated fitness costs and genetic drift. For realistic rates of dispersal between two neighbouring mosquito populations, it has been shown that the flow of transgenes from a targeted population is not likely to be sufficient to exceed the threshold in the neighbouring population [16, 17]. This implies that the UD gene drive system is unlikely to spread, at any significant frequency, into neighbouring regions. These results have been considered within a discrete population structure, however isolation-by-distance may be a more appropriate consideration for some populations. A number of previous studies have considered such a population structure in the context of an advantageous gene or chromosome rearrangements (e.g. [60–64]) but, to our knowledge, not in the context of two-locus gene drive systems such as those studied here. The existence of the threshold transgene frequency should also allow for the UD system to be recalled by introducing sufficient numbers of wild-type mosquitoes back into the population such that the transgene frequency falls below the threshold, thus causing the effect of the fitness cost to exceed the strength of the genetic drive mechanism.

Due to their self-limiting nature, KR systems may be viewed as an ideal system with which to test the effects of a given cargo gene before they are incorporated into self-sustaining systems. However, in spite of the fact that KR is inherently self-limiting, the same regulatory questions are still likely to need addressing. Modelling work has demonstrated that KR transgenes can spread into neighbouring populations at appreciable frequencies and then be eliminated due to their self-limiting nature. For example, the modelling work of Marshall & Hay [16] considered the case of a KR system with heterozygous and homozygous fitness costs of 2.5% and 5%, respectively for each allele and 1% migration between two neighbouring populations. When considering an initial transgene frequency of 0.5, maximum transgene frequencies of ∼0.95 and ∼0.3 were reached in the target and neighbouring populations, respectively. To our knowledge there have been no formal recall mechanisms detailed for KR systems as they should naturally diminish in time. However, releasing wild-type individuals into the population would lower the transgene frequency, and should result in more rapid transgene elimination.

Much of the previous modelling work on UD and KR systems has assumed a discrete (non-overlapping) generation framework in order to study the population genetics of these systems [8, 9, 15, 16, 18]. The works of Huang et al. [19, 20] considered the effects of age and spatial structure in a mosquito population in the context of the UD system. A large number of ecological factors have been included in studies of KR systems using the simulation model Skeeter Buster [21, 22], however the individual effects of each are not easily separated in such a complex model. Here we aim to further the understanding of how ecological factors affect the efficacy of UD gene drive systems and disentangle their effects from one another in the case of KR systems.

Here we begin by formulating population dynamics models of UD and KR gene drive systems that include a number of ecological factors such as the number of breeding sites; strength of density dependent larval competition; population growth rate; adult mortality rate; and rates of migration between two populations. These models also capture a number of characteristics of the control measures including the amount of mosquitoes released; the sexes released (release of males and females in 1:1 ratio (“bisex release”) or male-only release); fitness costs of introduced transgenes; sexes affected by lethal effects (bisex lethality or female-specific); whether one copy of a suppressor construct is sufficient to counter two copies of the related lethal construct; and the timing of the fitness/lethal effects (early- or late-acting). These models are first used to investigate the effects of the ecological factors considered here on the equilibrium size of a mosquito population in absence of control. We then go on to explore how such variation in mosquito population size (due to numbers of breeding sites) impacts upon UD or KR gene drive systems in terms of threshold transgene frequencies, relative degrees of population suppression and the time-scales of action. The strength of density dependent larval competition is then considered in terms of its effects on the observed dynamics of UD and KR systems as well as the thresholds for transgene introgression (UD) or increases in rescue transgene frequency (KR). Finally, using adjusted versions of the models we consider how rates of migration between a target and a non-target mosquito population can impact upon the efficacy of UD and KR gene drives and discuss results in terms of the desirability of observed outcomes. Whilst we present results for *Ae. aegypti* mosquito populations, we expect the models presented to be equally applicable for other species given a suitable parameterisation of the models.

## Materials and methods

### Combined population genetics and population dynamics model

Here we present a population dynamics model that may be used flexibly to describe both the UD and KR systems shown in Fig 1. With appropriate choices of lethality parameters, we anticipate this model could also be used to represent some alternative classes of gene drive such as *Medusa* [58] and reciprocal chromosome translocations [59]. We also believe this model to be both simple and general enough as to be equally applicable to other species. However, we restrict our attention to *Ae. aegypti* due to both the ongoing development of these systems and the availability of parameter values in the previous literature. This model is based on those of Yakob & Bonsall [23] and Alphey & Bonsall [24] in that it is a deterministic representation of a panmictic (randomly mating) population with continuous reproduction and a 1:1 male to female ratio both in the initial population and in the eggs laid in subsequent generations. Further, this model assumes that transgenic constructs do not display any sex linkage, will not mutate and the individual genetic components at a given locus will not separate. We also assume here that resistance alleles will not emerge within the population. In such systems, the introduction of two independently inherited transgenic constructs at unlinked loci results in a total of nine possible genotypes. Within this work these are referred to by a collection of four letters depending on the presence (*A* and *B*) or absence (*a* and *b*) of the two transgenic constructs and assigned a number (*i* = 1,…,9) in order to simplify notation within the mathematical model (see Table 1). For example, an individual that is heterozygous for both transgenic constructs will be denoted *AaBb* and assigned a genotype number *i* = 5.

**Table 1:** A table summarising the fitness and lethality parameters used throughout this study. Within the mathematical model numbers of each transgenic construct carried by a particular genotype (*i*) are denoted $\eta_{i}^{A}$ and $\eta_{i}^{B}$ for transgenic constructs A and B, respectively. Also shown within this table are the lethality parameters (*γ*_*i*_) used to define each of the gene drive elements with *γ* = 1 representing 100% effective lethality whilst *γ* = 0 denotes a viable genotype. Here the different gene drives considered are denoted UD (engineered underdominance) and KR (killer-rescue) with SS and WS representing strong and weak suppression of lethals, respectively. Here strong suppression of lethals refers to the scenario whereby one copy of a suppressor transgene is sufficient to rescue against two copies of the associated lethal transgene, whereas for weak suppression two suppressor copies are required to save against two lethal copies. For killer-rescue constructs, parameter values were selected such that construct *A* represents the killer and *B* the rescue.

		Constructs Carried		Lethal Effect (*γ*_*i*_)			
*i*	Genotype	$\eta_{i}^{A}$	$\eta_{i}^{B}$	UD (SS)	UD (WS)	KR (SS)	KR (WS)
1	*aabb*	0	0	0	0	0	0
2	*aaBb*	0	1	1	1	0	0
3	*aaBB*	0	2	1	1	0	0
4	*Aabb*	1	0	1	1	1	1
5	*AaBb*	1	1	0	0	0	0
6	*AaBB*	1	2	0	1	0	0
7	*AAbb*	2	0	1	1	1	1
8	*AABb*	2	1	0	1	0	1
9	*AABB*	2	2	0	0	0	0

We consider the overall fitness (Ω_*i*_) of each genotype (relative to wild-type individuals) to be composed of the lethal effects from transgenic constructs (*γ*_*i*_) and the fitness costs associated with the insertion of transgenes into the mosquito genome. Within the mathematical model these effects are captured by

$$\Omega_{i}^{M,F}=\epsilon_{A}^{\eta_{i}^{A}}\epsilon_{B}^{\eta_{i}^{B}}(1-\gamma_{i}^{M,F}),$$ (1)

where *ϵ*_*A*_ and *ϵ*_*B*_ denote the relative fitness of individuals carrying one copy of transgenic constructs *A* and *B*, respectively. These fitness effects manifest themselves as differences in the rates of survival of transgenic individuals relative to their wild-type counterparts. Note that the fitness cost associated with a particular choice of relative fitness parameter may be calculated as 1 − *ϵ*_*A*_ or 1 − *ϵ*_*B*_. These are applied multiplicatively such that, for example, an individual carrying two copies of construct *A* has relative fitness $\epsilon_{A}^{2}$ while a double heterozygote has relative fitness $\epsilon_{A}^{1}\epsilon_{B}^{1}$. For details on the number of copies of each transgenic construct carried by a given genotype see Table 1. Here a value of *ϵ* = 1 means an individual is as fit as a wild-type individual whereas *ϵ* = 0 represents a completely non-viable individual. The other component of an individual’s overall fitness is the lethal effect of the carried transgenes (*γ*_*i*_). In this case *γ*_*i*_ = 0 means that individuals of genotype *i* are 100% viable whereas individuals with *γ*_*i*_ = 1 receive a 100% lethal effect. Note that some systems may exhibit incomplete penetrance of lethal transgenes (i.e. 0 < *γ*_*i*_ < 1), however this is beyond the scope of this study. Parameter values describing the lethal effects of transgenes on each genotype in UD and KR systems are summarised in Table 1. Note that as in a previous population genetics study of UD [18] we here consider the possibility that different numbers of suppressor copies may be required to nullify the effect of the associated lethals. These are termed a “strongly suppressed” system when one suppressor copy is sufficient to nullify two copies of a given lethal or a “weakly suppressed” system where two suppressor copies are required to nullify the effect of two lethal copies.

It is now possible to outline a set of equations describing the dynamics of each mosquito genotype in time. As with the work of Alphey & Bonsall [24], the models presented here are adaptations of Kostitzin’s [33] work that used Lotka-Volterra type equations to represent competition between different genotypes. These models also consider a nonlinear representation of intraspecific competition due to Maynard Smith & Slatkin [34] that can describe a wide range of density dependence scenarios [35]. We also consider two sets of equations representing distinct scenarios in terms of the developmental stage at which fitness/lethal effects of transgenic constructs act. In particular, we consider the cases where these effects are either “early-acting”; taking effect before density-dependent competition (e.g. in eggs/early instar larvae) or “late-acting”; having an impact after density-dependent competition and before mating (eg. in pupae or pharate adults). This results in the following equations

**Early-acting fitness/lethal effects**:

$$\frac{dM_{i}\left(t\right)}{dt}=\frac{\rho v_{i}(t-\tau )\Omega_{i}^{M}}{1+(\sum_{i\in \sigma }\alpha [v_{i}(t-\tau )\Omega_{i}^{M}+w_{i}(t-\tau )\Omega_{i}^{F}]{)}^{\beta }}-\mu M_{i}\left(t\right),$$ (2)

$$\frac{dF_{i}\left(t\right)}{dt}=\frac{\rho w_{i}(t-\tau )\Omega_{i}^{F}}{1+(\sum_{i\in \sigma }\alpha [v_{i}(t-\tau )\Omega_{i}^{M}+w_{i}(t-\tau )\Omega_{i}^{F}]{)}^{\beta }}-\mu F_{i}\left(t\right),$$ (3)

**Late-acting fitness/lethal effects**:

$$\frac{dM_{i}\left(t\right)}{dt}=\frac{\rho v_{i}(t-\tau )\Omega_{i}^{M}}{1+(\sum_{i\in \sigma }\alpha [v_{i}(t-\tau )+w_{i}(t-\tau )]{)}^{\beta }}-\mu M_{i}\left(t\right),$$ (4)

$$\frac{dF_{i}\left(t\right)}{dt}=\frac{\rho w_{i}(t-\tau )\Omega_{i}^{F}}{1+(\sum_{i\in \sigma }\alpha [v_{i}(t-\tau )+w_{i}(t-\tau )]{)}^{\beta }}-\mu F_{i}\left(t\right).$$ (5)

Note that these two models are identical except for the removal of the fitness/lethal effect parameter (Ω) from the denominator of the late-acting model since these effects do not act until after the density dependent phase. A limitation of such models is that density dependent competition may only occur between larvae born at the same time. Definitions of parameters and variables are given in Table 2 alongside a base set of parameter values used throughout this study. Expressions for *v*_*i*_ and *w*_*i*_ are outlined in S1 Appendix Section 1 and S1 Table alongside details regarding the implementation of release ratios (and therefore initial conditions) and transgene frequency calculations.

**Table 2:** A table of definitions and typical values for each parameter and variable used within the model.

Symbol	Description	Value	Source
*N*(*t*)	Total adult mosquitoes	$N\left(t\right)=\sum_{i=1}^{9}(M_{i}\left(t\right)+F_{i}\left(t\right))$	[25, 26]
*M*_*i*_(*t*)	Adult males of genotype *i*	——	
*F*_*i*_(*t*)	Adult females of genotype *i*	——	
*v*_*i*_(*t*)	Male eggs/immatures of genotype *i* resulting from mating of the existing adult population	——	
*w*_*i*_(*t*)	Female eggs/immatures of genotype *i* resulting from mating of the existing adult population	——	
*α*	Density parameter (1/*α* is related to number of available breeding sites)	0.02 (chosen to produce realistic population sizes)	
*β*	Strength of density-dependent competition	1.10	[27, 28]
*ρ*	Per-capita population growth rate (net of density-independent survival through immature stages)	1.01 day^−1^	[29]
*μ*	Adult mortality rate	0.12 day^−1^	[27, 30, 31]
*τ*	Time delay (egg—adult generation time)	18 days	[32]
*σ*	Set of all possible genotypes	{1,2,3,4,5,6,7,8,9}	
*ϵ*_*A*_	Relative fitness of individuals with one copy of construct A	0 ≤ *ϵ*_*A*_ ≤ 1	
*ϵ*_*B*_	Relative fitness of individuals with one copy of construct B	0 ≤ *ϵ*_*B*_ ≤ 1	
$\eta_{i}^{A}$	Number of copies of construct A	0, 1 or 2	
$\eta_{i}^{B}$	Number of copies of construct B	0, 1 or 2	
$\gamma_{i}^{M,F}$	Lethality to males (*M*) or females (*F*) of genotype *i*	0 or 1	
$\Omega_{i}^{M,F}$	Total relative fitness of males (*M*) or females (*F*) of genotype *i*	$\Omega_{i}^{M,F}=\epsilon_{A}^{\eta_{i}^{A}}\epsilon_{B}^{\eta_{i}^{B}}(1-\gamma_{i}^{M,F})$	
*θ*	Release ratio	*θ* = Introduced/Wild	
*ψ*	Rate of migration between populations	Various (Day^−1^)	

### Incorporating migration

**Fig 2:**
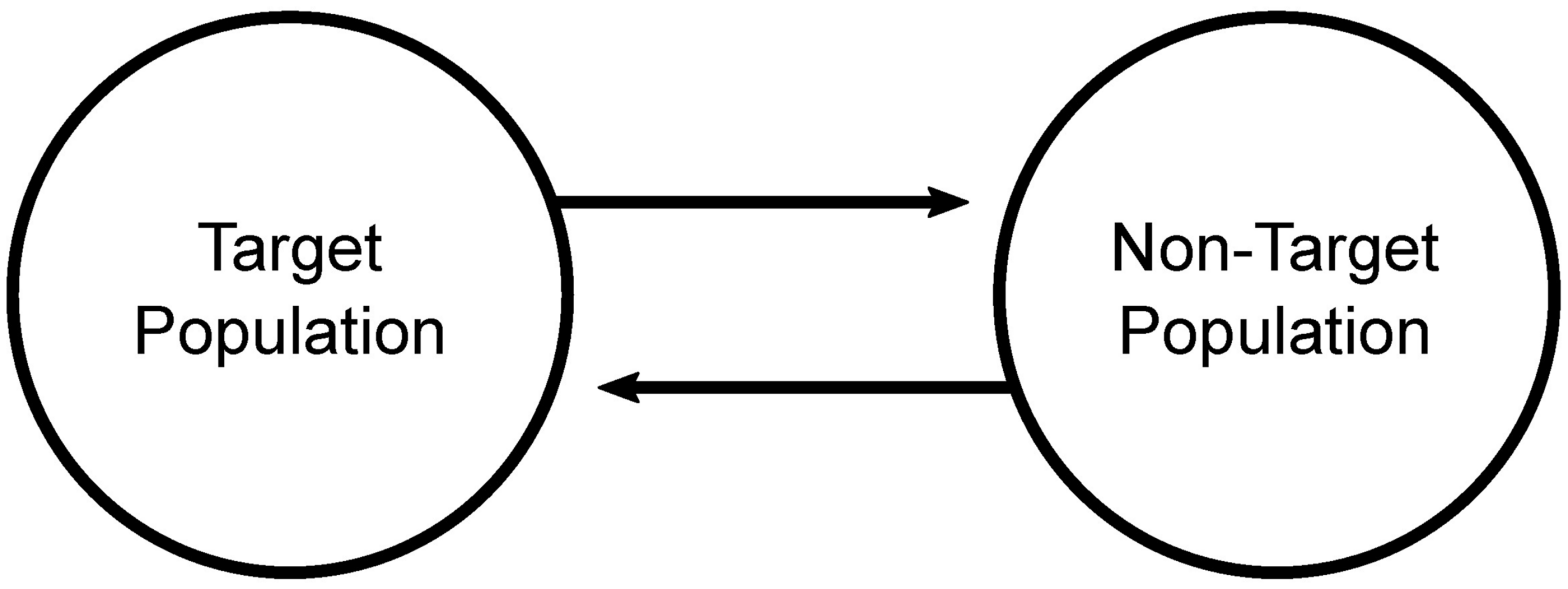
After [36], a schematic representation of the two-deme model. Here a separate population dynamics model is considered for each of the target population and non-target population. These are then linked by two-way migration. This is modelled as a continuous transfer of individuals between the two demes at a rate *ψ* which is expressed per day. Simply, each of the two demes continuously swap a fraction of their population with the other, thus the total amount of migration will depend on the size of each population. Whilst the rates of migration per day considered within this study may appear very low, they actually add up to quite high fractions of the overall populations being exchanged in each generation (comparable to values used in other studies [16, 36, 37]).

As discussed above, one of the key concerns associated with gene drive systems is the degree to which they might affect non-target, neighbouring populations. In order to assess how population dynamics can impact upon the ability of a system to spread into a neighbouring population, we formulate here a two-deme population dynamics model (Fig 2). This is used to investigate both the spread of transgenes into the neighbouring population and changes (lasting or transient) in the overall size of a neighbouring population. Two-deme models such as this are well established in the literature for population genetics models of gene drive systems (for example [16, 36, 37]) but these models rarely incorporate population dynamics in addition to genetics. We considered that for gene drive systems predicted to cause significant excess mortality, consideration of population dynamics might be important.

The consideration of two demes requires some modifications to be made to the equations stated in the previous section. Firstly, since we are now modelling the dynamics of two populations, we must consider an extra set of delay-differential equations representing the second (non-target) population. These take the same form as those given in the previous section except for the incorporation of migration between the two demes. This is achieved through the addition of extra terms into each equation which are of the form $\psi (M_{i}^{O}\left(t\right)-M_{i}^{T}\left(t\right))$ for the target population and $\psi (M_{i}^{T}\left(t\right)-M_{i}^{O}\left(t\right))$ for the non-target population. Here the superscripts *T* and *O* denote the target and non-target populations, respectively. Similar terms are also included for female populations but with *M* replaced by *F*. This gives models of the form

**Early-acting fitness/lethal effects**:

$$\frac{dM_{i}^{T}\left(t\right)}{dt}=\frac{\rho v_{i}^{T}(t-\tau )\Omega_{i}^{M}}{1+(\sum_{i\in \sigma }\alpha [v_{i}^{T}(t-\tau )\Omega_{i}^{M}+w_{i}^{T}(t-\tau )\Omega_{i}^{F}]{)}^{\beta }}-\mu M_{i}^{T}\left(t\right)+\psi \left(M_{i}^{O}\left(t\right)-M_{i}^{T}\left(t\right)\right),$$ (6)

$$\frac{dF_{i}^{T}\left(t\right)}{dt}=\frac{\rho w_{i}^{T}(t-\tau )\Omega_{i}^{F}}{1+(\sum_{i\in \sigma }\alpha [v_{i}^{T}(t-\tau )\Omega_{i}^{M}+w_{i}^{T}(t-\tau )\Omega_{i}^{F}]{)}^{\beta }}-\mu F_{i}^{T}\left(t\right)+\psi \left(F_{i}^{O}\left(t\right)-F_{i}^{T}\left(t\right)\right),$$ (7)

$$\frac{dM_{i}^{O}\left(t\right)}{dt}=\frac{\rho v_{i}^{O}(t-\tau )\Omega_{i}^{M}}{1+(\sum_{i\in \sigma }\alpha [v_{i}^{O}(t-\tau )\Omega_{i}^{M}+w_{i}^{O}(t-\tau )\Omega_{i}^{F}]{)}^{\beta }}-\mu M_{i}^{O}\left(t\right)+\psi \left(M_{i}^{T}\left(t\right)-M_{i}^{O}\left(t\right)\right),$$ (8)

$$\frac{dF_{i}^{O}\left(t\right)}{dt}=\frac{\rho w_{i}^{O}(t-\tau )\Omega_{i}^{F}}{1+(\sum_{i\in \sigma }\alpha [v_{i}^{O}(t-\tau )\Omega_{i}^{M}+w_{i}^{O}(t-\tau )\Omega_{i}^{F}]{)}^{\beta }}-\mu F_{i}^{O}\left(t\right)+\psi \left(F_{i}^{T}\left(t\right)-F_{i}^{O}\left(t\right)\right),$$ (9)

**Late-acting fitness/lethal effects**:

$$\frac{dM_{i}^{T}\left(t\right)}{dt}=\frac{\rho v_{i}^{T}(t-\tau )\Omega_{i}^{M}}{1+(\sum_{i\in \sigma }\alpha [v_{i}^{T}(t-\tau )+w_{i}^{T}(t-\tau )]{)}^{\beta }}-\mu M_{i}^{T}\left(t\right)+\psi \left(M_{i}^{O}\left(t\right)-M_{i}^{T}\left(t\right)\right),$$ (10)

$$\frac{dF_{i}^{T}\left(t\right)}{dt}=\frac{\rho w_{i}^{T}(t-\tau )\Omega_{i}^{F}}{1+(\sum_{i\in \sigma }\alpha [v_{i}^{T}(t-\tau )+w_{i}^{T}(t-\tau )]{)}^{\beta }}-\mu F_{i}^{T}\left(t\right)+\psi \left(F_{i}^{O}\left(t\right)-F_{i}^{T}\left(t\right)\right),$$ (11)

$$\frac{dM_{i}^{O}\left(t\right)}{dt}=\frac{\rho v_{i}^{O}(t-\tau )\Omega_{i}^{M}}{1+(\sum_{i\in \sigma }\alpha [v_{i}^{O}(t-\tau )+w_{i}^{O}(t-\tau )]{)}^{\beta }}-\mu M_{i}^{O}\left(t\right)+\psi \left(M_{i}^{T}\left(t\right)-M_{i}^{O}\left(t\right)\right),$$ (12)

$$\frac{dF_{i}^{O}\left(t\right)}{dt}=\frac{\rho w_{i}^{O}(t-\tau )\Omega_{i}^{F}}{1+(\sum_{i\in \sigma }\alpha [v_{i}^{O}(t-\tau )+w_{i}^{O}(t-\tau )]{)}^{\beta }}-\mu F_{i}^{T}\left(t\right)+\psi \left(F_{i}^{O}\left(t\right)-F_{i}^{O}\left(t\right)\right),$$ (13)

within which *ψ* represents the rate of migration while the definitions of other parameters and variables remain unchanged. Note that migration terms do not include any developmental time delays since this is assumed to comprise adult mosquitoes moving from one deme to the other prior to mating on a given day. Here we have excluded from our model the possibility of human-mediated movement of mosquito eggs as this is likely to be negligible relative to adult movement on the spatial scale considered here. It is also worth noting that models not including migration are a special case of those presented here with the rate of migration set to zero (i.e. *ψ* = 0). All numerical simulations within this study are created using MATLAB (The MathWorks Inc., Natick, MA) delay differential equation solver dde23 [38].

## Results

### Effects of ecological parameters on overall population size

The mathematical models outlined in the previous section contain a number of ecological parameters, namely the carrying capacity, primarily based on the number and quality of breeding sites (i.e. carrying capacity is proportional to 1/*α*); the strength of density-dependent larval competition (*β*); the intrinsic per capita rate of population growth (*ρ*); and the adult mortality rate (*μ*). Before investigating the impact of such ecological parameters on the efficacy of UD and KR gene drive systems, we begin by studying their effects on the overall size of an isolated mosquito population in absence of genetic control. This can be investigated by considering the steady-state equation defined by Alphey & Bonsall [24], i.e.

$$N^{*}=\frac{1}{\alpha }(\frac{\rho }{\mu }-1{)}^{1/\beta },$$ (14)

where N* denotes the equilibrium population size in absence of control.

**Fig 3:**
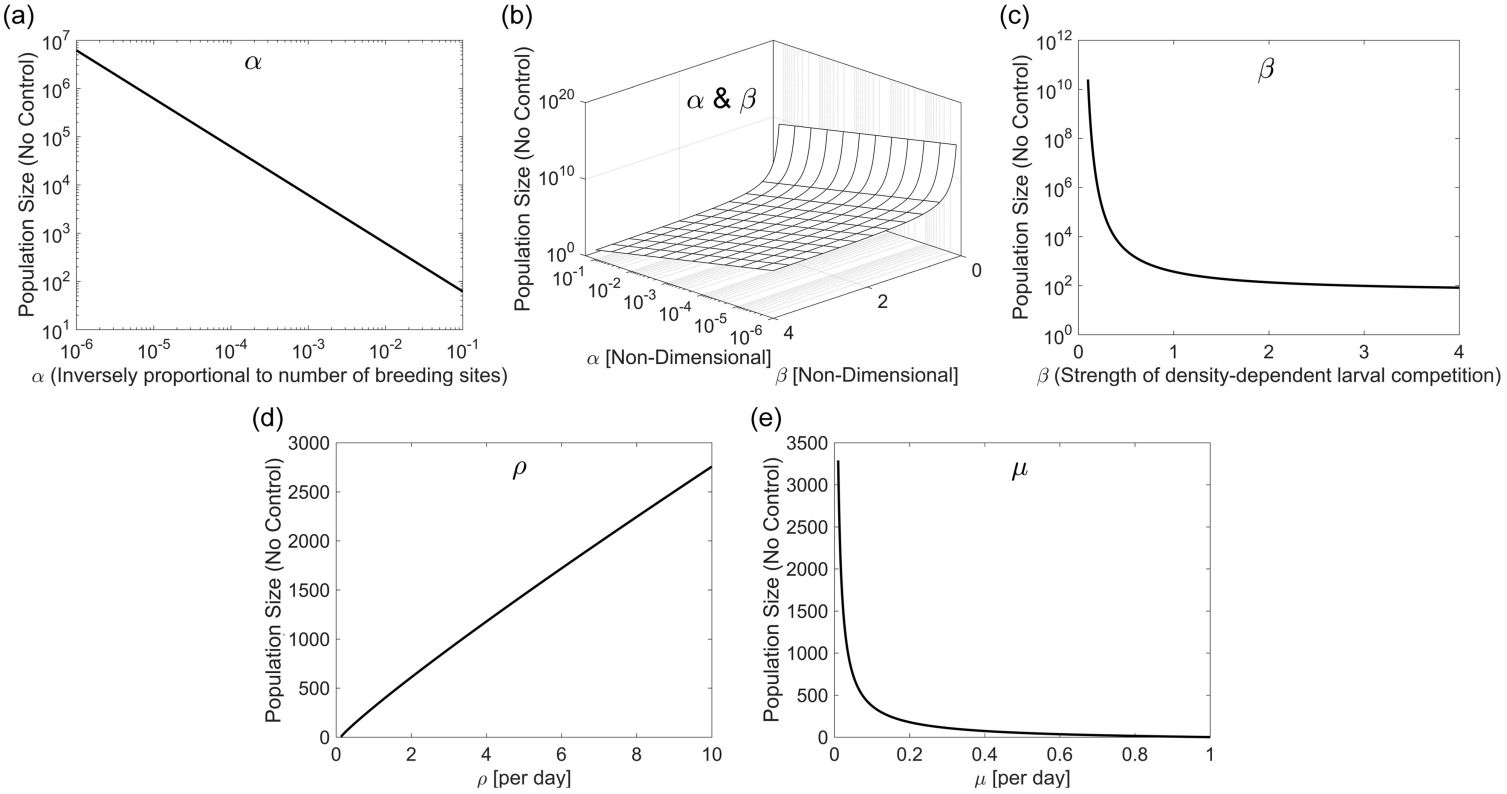
Ecological parameters influence the size of a mosquito population in absence of any control measures. Individual panels here show the effect on the mosquito population size of varying (a) *α*, representing the carrying capacity of the environment (eg. availability of larval habitats); (c) strength of density-dependent larval competition; (d) the intrinsic per capita population growth rate; and (e) adult mortality rate whilst the other three are held constant at a base value (see Table 2). Panel (b) shows the combined effect on the population size from varying both the availability of larval habitats and the strength of density dependence. Note that panels (a), (b) and (c) use logarithmic axes to aid in visualisation of results.

We now investigate the effects of each parameter on the overall equilibrium population size by considering variations in each while the other three are held constant—at the base values detailed in Table 2. Since little is known about these ecological parameters in wild populations we vary each over a range wide enough that it is likely to span both realistic and biologically unfeasible scenarios. This gives results as shown in Fig 3.

These results clearly show that, in a mathematical model of this type, each of the four ecological parameters are able to significantly affect the equilibrium population size. We see from Fig 3(a) and 3(c) that parameters related to density-dependent larval competition (i.e. *α* and *β*) have the largest impact on the overall population size. The intrinsic per capita rate of population growth (*ρ*) and the adult mortality rate (*μ*) are also capable of altering the overall equilibrium population size, but over a much smaller range than is possible with *α* and *β*. It is clearly possible here for variations in these parameters to result in very large or small population sizes. It is expected that these results span both realistic and biologically non-feasible population sizes. Combining mathematical models and data on population sizes (e.g. [39]) can help to narrow down the realistic parameter range, however further experimental data is required to refine these yet further.

### What effect does population size have?

The previous section explored the effects of various ecological parameters on the overall size of a mosquito population. We now examine whether such changes in the overall size of a population before the release of transgenic mosquitoes will impact upon the outcome or dynamics of UD and KR gene drive releases. We explore this in the context of a number of important performance metrics. In order to explore these effects we simulate a 1:1 (introduced:wild) release of each gene drive system. This release is modelled as the introduction of *AABB* genotype adults at a ratio equal to the number of wild-type adult mosquitoes (at equilibrium) at the time of release (see Supplementary Information Section 1 for further details). We explore this release strategy over the full range of relative fitness parameters (i.e. 0 ≤ *ϵ*_*A*/*B*_ ≤ 1) and for a number of different initial mosquito population sizes.

For changes in the initial population size, we consider only changes to the availability of larval habitats within an environment (proportional to 1/*α*). Specifically, we consider eight different initial population sizes spanning five orders of magnitude (10^0^ to 10^4^), corresponding to *α* values of 0.7, 0.2, 0.07, 0.02, 0.007, 0.002, 0.0007 and 0.0002 (see Fig 3(a)). To explore whether these changes in population size alter the behaviour of the systems studied here, we consider a number of metrics that represent different aspects of their performance. For the UD system we consider three performance metrics, namely the change in equilibrium population size; the maximal degree of transient population suppression; and the time taken for the system to reach maximum population suppression. For the KR system we consider the same metrics except there will be no equilibrium change in the population size since the system is self-limiting. In each case we run numerical simulations for each of the eight *α* values and repeat this for values of *ϵ* (relative fitness) spanning the entire feasible range (i.e. 0 ≤ *ϵ* ≤ 1). Results obtained here indicate that no behavioural differences result from variation in the initial population size, so long as the release ratio is held constant (i.e. the absolute release size must be adjusted relative to the overall population).

**Fig 4:**
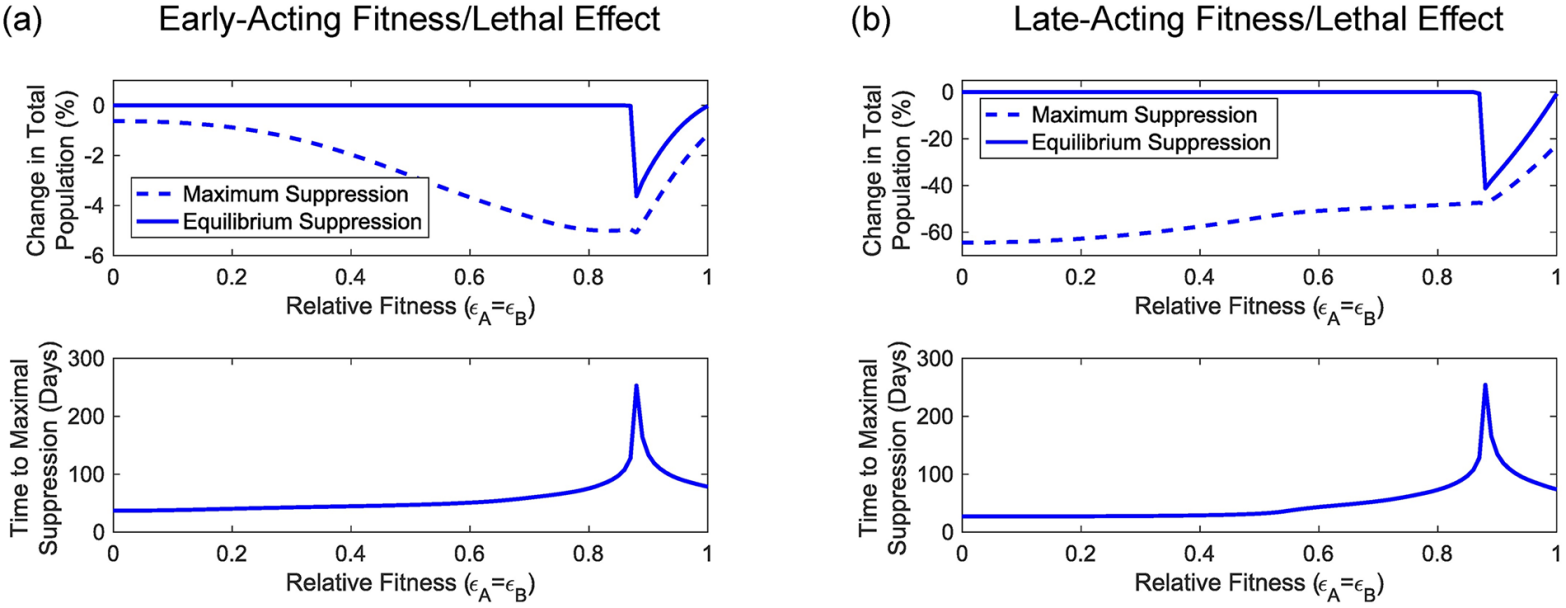
Initial adult mosquito population sizes have no impact on a number of key performance indicators for two-locus engineered underdominance gene drive. Panels (a) and (b) show the impacts of early- and late-acting fitness/lethal effects whilst the different lines represent the general pattern for a given indicator. The top row shows both the maximal and equilibrium amount of population suppression for a two-locus engineered underdominance gene drive system whereas the bottom row shows the time taken for a system to reach maximal population suppression. We find no difference between examples for different values of *α*. As such, lines are results from a set of numerical simulations spanning the full range of relative fitness parameters (i.e. 0 ≤ *ϵ*_*A*,*B*_ ≤ 1) for a randomly selected *α*.

From the results of Fig 4 we identify a number of interesting features of the UD gene drive system. Firstly, similar to previous results for other classes of gene drive (e.g. [24, 30]) we find significant differences between systems that use early-acting as opposed to late-acting transgenes. Late-acting transgenes cause greater amounts of both maximal and equilibrium population suppression (∼65% and ∼40%, respectively) than the early-acting system that produces just ∼5% and ∼4%, respectively when considering a 1:1 (introduced:wild) release ratio. However, we do not see any large differences in either the time taken to reach the maximal level of population suppression or the threshold fitness costs that produce lasting transgene introgression between the early and late acting systems. The region with no equilibrium population suppression below *ϵ*_*A*_ = 0.88 = *ϵ*_*B*_ is due to the elimination of the transgenes, but above this we see an amount of suppression that decreases as transgenic individuals become more fit (i.e. as *ϵ*_*A*_ = *ϵ*_*B*_ → 1).

**Fig 5:**
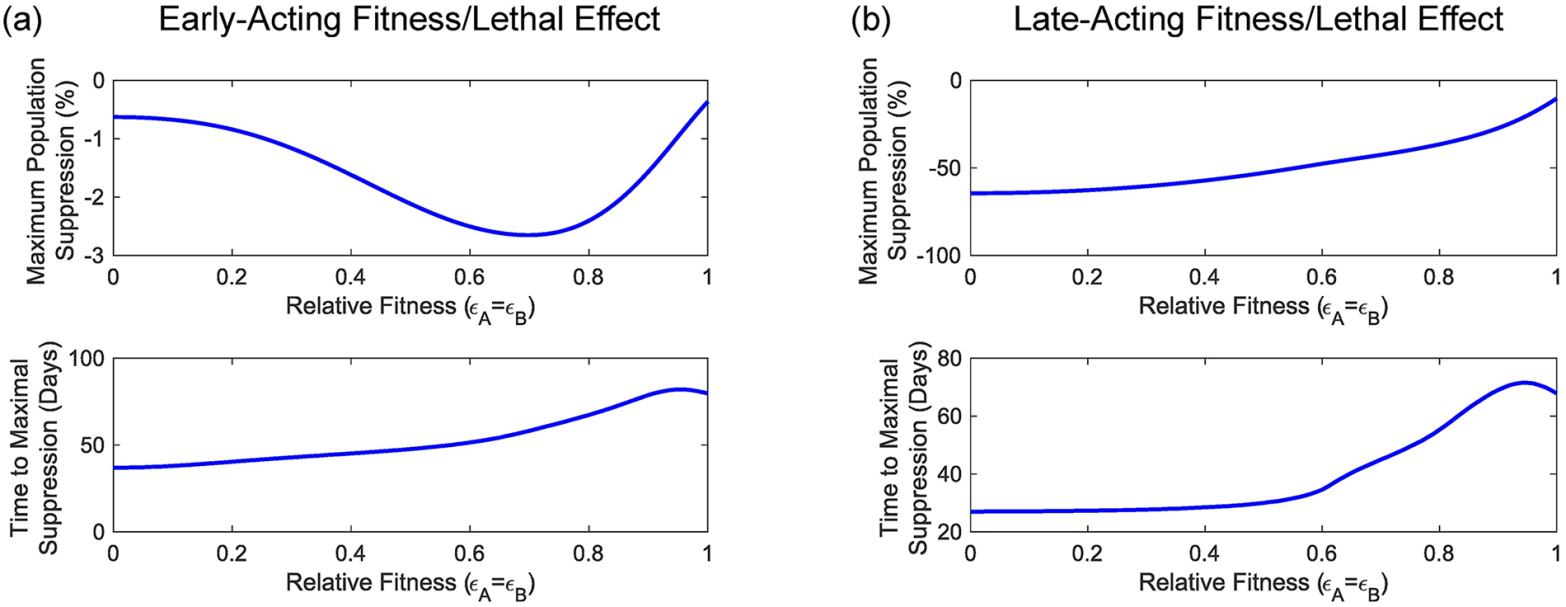
Initial adult mosquito population sizes have no impact on two performance indicators for the killer-rescue gene drive system. Panels (a) and (b) show the impacts of early- and late-acting fitness/lethal effects, respectively. The lines represent a general pattern for a given indicator. The top row of plots show the maximum degree of population suppression given by a killer-rescue system whilst the bottom row of plots show the time-taken for the system to reach this level. We find no difference between examples for different values of *α*. As such, lines are results from a set of numerical simulations spanning the full range of relative fitness parameters (i.e. 0 ≤ *ϵ*_*A*,*B*_ ≤ 1) for a randomly selected value of *α*.

For the KR system, the results in Fig 5 show that late-acting systems cause much greater levels of population suppression than early-acting ones. In particular, for the release ratio considered here (1:1, introduced:wild) we see that a late-acting system causes at most ∼65% population suppression whereas the early-acting system produces just ∼2.5%. We also observe a significantly different pattern of suppression as relative fitness varies. Unlike the UD system, here we observe sizeable differences in the time taken to reach the maximum level of population suppression. The early-acting system has a greatest time to maximal population suppression of ∼80 days whilst the late-acting system can take up to ∼70 days. We also observe a difference between early and late acting systems in terms of the relationship between relative fitness parameters and the time taken to reach maximal population suppression.

The differences observed here in levels of population suppression between early- and late-acting systems have been explained previously for other gene drive systems [24, 30]. For early-acting systems, fitness/lethal effects act to lower larval density, thereby reducing the density dependent competition experienced later and partially compensating for the effects of introduced transgenes. For late-acting systems, fitness/lethal effects act after density-dependent competition, providing an extra round of suppression that cannot be compensated for. Thus, late-acting systems produce a greater degree of population suppression.

Whilst we observe significant differences in the degrees of population suppression caused by early- and late-acting UD and KR systems, we notice only tiny variations due to changing the initial population size. In particular, for each panel in Figs 4 and 5, some coloured symbols (representing results with different initial population sizes) display extremely small deviations from the grey lines (randomly chosen sample simulations). These deviations are likely to represent differences in numerical error between the eight examples, as explained in S1 Appendix Section 2, S1 and S2 Figs. This suggests that each system will produce consistent behaviour in terms of time scale, genotype frequencies and population suppression for any initial population size (see S3 Fig for time courses showing population size effects for the full range of relative fitness parameters studied here). We would expect this result to hold true so long as the population size is large enough to avoid stochastic and Allee effects (which are not included in this model).

### Density-dependent larval competition can produce a range of different behaviours

**Fig 6:**
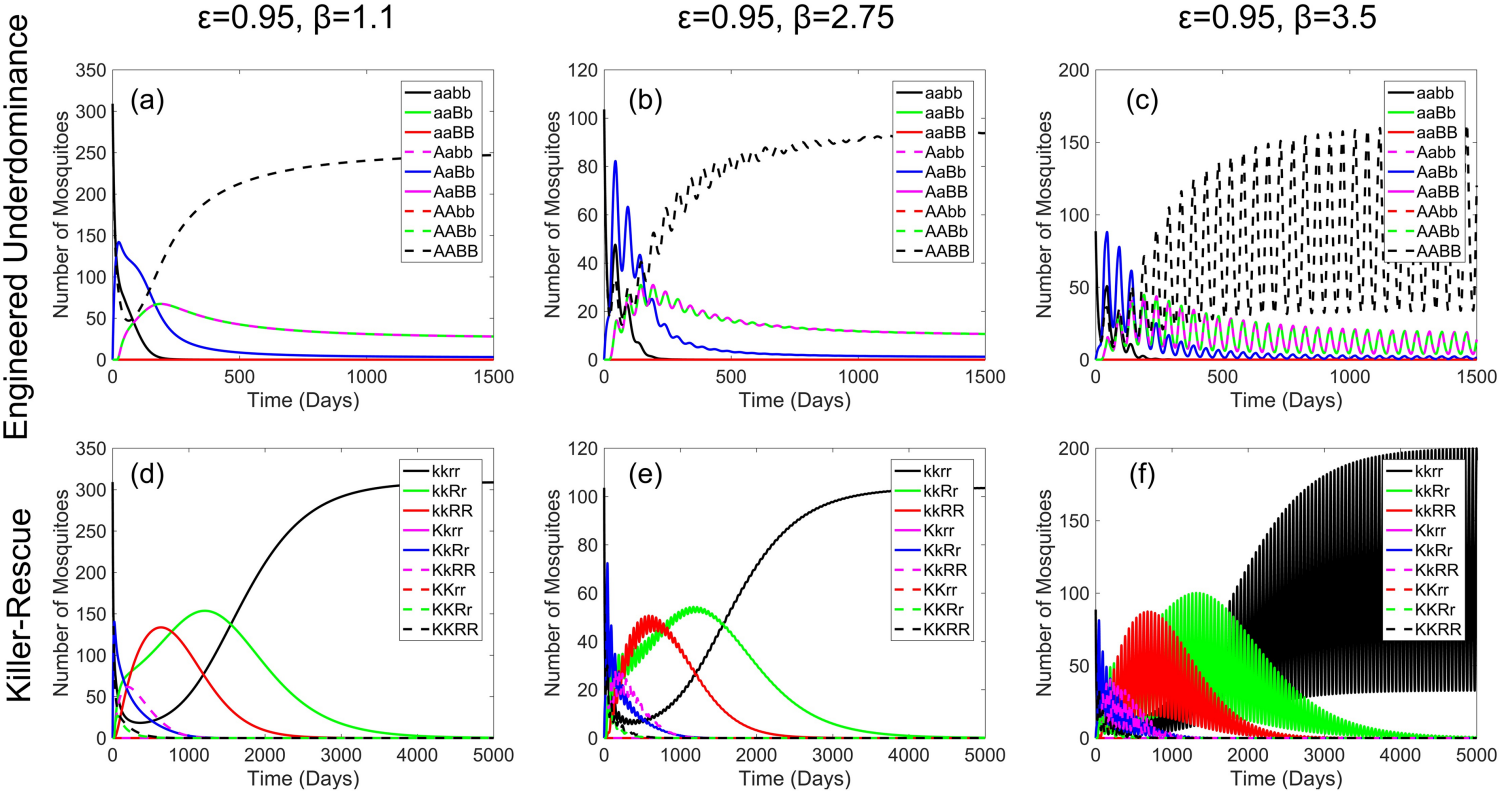
Plots showing examples of the range of behaviour attainable from population dynamics models of engineered underdominance and killer-rescue gene drive systems with early-acting lethals. Here the top row of panels ((a)-(c)) show effects in the engineered underdominance system whereas the bottom row ((d)-(f)) demonstrate effects in the killer-rescue system. Columns display different degrees of density dependence. From left to right these are weak overcompensatory ((a) and (d), *β* = 1.1); intermediate overcompensatory ((b) and (e), *β* = 2.75); and strong overcompensatory ((c) and (f), *β* = 3.5) dynamics. Initial wild-type adult population sizes with these strengths of density dependence are ∼309 (for *β* = 1.1), ∼104 (for *β* = 2.75) and ∼89 (for *β* = 3.5). For *β* = 1.1 the system displays stable dynamics whereas for *β* = 2.75 and *β* = 3.5 we see oscillatory dynamics that are damped and neutral, respectively. Assuming the cargo (refractory) gene is fully effective in a single copy, it is important to consider here the number of wild-type mosquitoes (solid black lines) relative to all others since these are the only genotype in which females are capable of transmitting viruses. Note here the differences in scaling of both the *x* and *y* axes.

Laboratory and field studies have suggested that the larval stages of mosquito development may be subject to some degree of density-dependent competition [27, 28, 40–48]. This often manifests itself as a reduction in the proportion of larvae that survive to adulthood in more densely packed environments [27, 42, 48]. A number of mechanisms have been proposed to explain why this may occur [28, 40, 49] yet, to our knowledge, none of these has yet been conclusively shown. Also subject to some degree of doubt is the strength of this density dependent effect. Some studies have suggested that mosquito populations are subject to only a very small density dependent effect [27], while others suggest that their dynamics may be strongly overcompensatory [46]. Since Alphey & Bonsall [24] demonstrated the potential for density dependent effects to result in oscillatory dynamics in the context of endonuclease-based gene drive systems, here we explore the potential range of behaviour that may occur as a result of varying the strength of density dependent larval competition in mosquito populations when either a UD or a KR system is introduced. While results are presented in Fig 6 for an *Ae. aegypti* population, we anticipate that similar results will also hold under alternative parameterisations representing other species.

Results for each system under a range of different *β* parameter values are given in Fig 6. Transgene frequencies obtained for each of the numerical simulations here are given in S4 Fig (see S1 Appendix Eqs (S3) and (S4) for calculations). In these simulations it is assumed that each construct carries a fitness cost of 5% per construct (i.e. *ϵ*_*A*_ = 0.95 = *ϵ*_*B*_); lethal genetic elements are strongly suppressed (i.e. one copy of the rescue element is sufficient); and the fitness/lethal effects are early-acting (i.e. prior to density dependent competition).

Fig 6 shows that altering the strength of density dependence does not lead to any major differences in the outcomes of the considered control measures either in terms of which type of individuals are eliminated (wild-type or transgenic) or the time taken for this to occur. This final outcome differs for the two systems modelled. For the UD system each example results in lasting transgene introgression whereas, for the KR system, the rescue transgene frequency (see S1 Appendix Eq (S4)) increases to very similar levels before declining toward elimination in a similar time frame. Additionally, each of the three strengths of density dependence considered in Fig 6 result in approximately equal maximum transgene frequencies and times to transgene elimination for each system (see S4 Fig). However, it is clear that whilst density dependence does not play a significant role in deciding the outcome of a given control strategy, it does play a major role in determining the dynamics observed in reaching that outcome. For example, consistent with results in the literature we observe stable, damped oscillatory or persistent oscillatory behaviour for different *β* values (as seen in Fig 6) [24, 27]. However, here the *β* thresholds for each of these behaviours differ in value from those identified in other studies. This is likely due to differences between these studies in terms of the inclusion of a developmental delay (not included in Dye [27]) and the difference in the length of this delay between *Ae. aegypti* and *Anopheles gambiae* (for Alphey & Bonsall [24]). For a more thorough stability analysis of this type of model see the Supplementary Information of Alphey & Bonsall [24].

**Fig 7:**
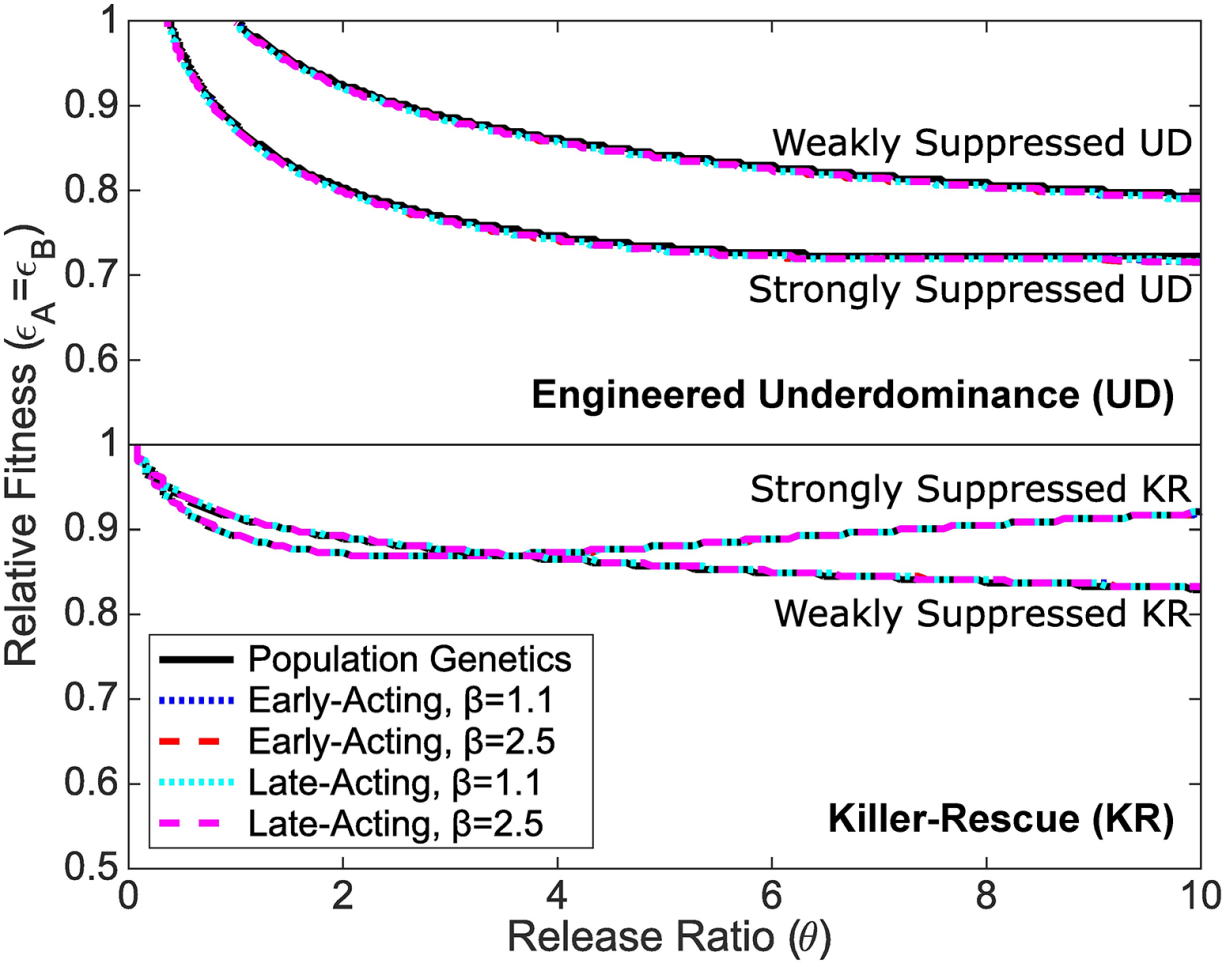
Plot showing comparisons between population genetics models [18] and population dynamics models in two density dependence scenarios (*β* = 1.1 and *β* = 2.5). The top two threshold lines are for lasting introgression of transgenes in the UD system with strongly and weakly suppressed lethals. The bottom two threshold lines are for increases in transgene frequency of the KR system with strongly and weakly suppressed lethals. The key shows the different scenarios plotted, however these are essentially super-imposed. Outcomes for weakly suppressed and strongly suppressed systems are different, but within those cases neither early versus late acting lethality nor varying beta (1.1 or 2.5) makes a significant difference on the thresholds plotted.

To test the idea that the strength of density dependence does not affect the outcome of UD and KR gene drives further we compare thresholds for lasting transgene introgression (UD) and increases in the rescue frequency (KR) under differing strengths of density dependent competition (*β*). Specifically, we discretise parameter space (release ratio (*θ*) and relative fitness (*ϵ*_*A*_ = *ϵ*_*B*_)), simulate the model system under each parameter configuration and extract the relevant threshold. The results in Fig 7 show comparisons of five data sets in each case. These are from population genetics models [18] and four data sets from the population dynamics model described here. These represent the possible combinations of early or late acting fitness/lethal effects with weakly (*β* = 1.1) or intermediately (*β* = 2.5) overcompensatory density dependence. These particular *β* values are chosen so as to be different enough to produce an observable difference and yet avoid *β* values producing oscillatory behaviour.

Fig 7 shows that the threshold lines from population genetics and population dynamics models lie extremely close together, suggesting that the strength of density dependence makes little or no difference to conditions for lasting transgene introgression (for UD) or increasing transgene frequency (for KR). Under a large degree of magnification (S11 Fig), small differences are visible, with increasing strength of density dependence having opposite effects on the two differing systems. For the UD system, increasing the strength of density dependence appears to very slightly raise the tolerable fitness costs conferred by transgenic constructs, however, for the KR system the same increase results in a very slight decrease in tolerable fitness costs. While this seems to imply a fundamental difference between the two systems, it is possible that this simply results from the necessity of using different criteria to define success in the two systems. The same results also demonstrate that, for a given strength of density dependence, the timing of the fitness/lethal effect appears to have little/no impact on the threshold lines displayed in Fig 7. The differences in tolerable fitness costs observed here are extremely small and so they are unlikely to materially alter the outcome of an implemented gene drive strategy. This is because any realistic gene drive release would likely aim to lie comfortably inside of the threshold lines of Fig 7 in order to ensure successful use [18].

### Effects on a non-target neighbouring population

Here we consider the effects of various different model/system configurations (sexes released, strength of lethal suppression and sexes affected by lethal genes) on the potential outcomes from a released gene drive on both a target and a non-target population when there is migration between the two. This is examined using the two-deme population dynamics model summarised in the Methods section. Previous work focusing on population genetics models has shown that UD systems are unlikely to spread into a neighbouring population at any significant frequency since they are unlikely to exceed the necessary threshold introduction frequency [16, 17]. By contrast, the same studies have shown that KR systems are capable of reaching significant frequencies in neighbouring populations but that these will not persist due to the self-limiting nature of the system [16].

Population genetics models have previously been used to study the migration of single-locus underdominant alleles and chromosome rearrangements [64–66]. Further to this, the effects of different gene drive classes have also been studied with population genetics models [16, 17, 36, 67]. In terms of two-locus UD systems, Marshall [17] and Marshall & Hay [16] demonstrated that these systems display thresholds for fixation and loss of transgenes. Specifically, they find a threshold migration rate of 4.3% per generation is necessary for a UD system with additive fitness costs of 5% (dominance 50%) to invade a neighbouring population. For the same fitness costs, they also demonstrate that a migration rate of 1% per generation, produces near-fixation of transgenes in the target population and a maximum frequency of ∼3.2% in a neighbouring population. To our knowledge, these are the only studies considering migration of UD systems between two neighbouring populations. However, they do not consider the effects of migration in terms of its impact on either wild-type or total absolute population sizes. As such, here we discretise a region of parameter space (relative fitness and rate of migration) and simulate both classes of gene drive, measuring different outcomes relevant to the respective systems.

As in the previous section, the differing nature of the two systems considered necessitates the use of different measures to fully explore their behaviour. In the case of the UD system we record the total population size and number of wild-type females in both the target and non-target population at the new equilibrium. For the KR system we consider different measures since the system eventually returns to the original equilibrium. In particular, we record the maximum and minimum (i.e. most suppressed) population sizes; maximum and minimum (i.e. most suppressed) wild-type female population sizes; and the maximum frequency attained by the ‘rescue’ transgene (calculated as per S1 Appendix Eq (S4)). Here we consider just the number of wild-type female mosquitoes to be important since they are the only individuals capable of transmitting viruses (assuming cargo genes are fully effective in a single copy). We also consider total population sizes so as to ensure that, within the parameter range considered, transgenic mosquito releases will not significantly increase the total population size and thus the rate of nuisance biting (or potential transmission (by females) of other mosquito-borne diseases).

**Fig 8:**
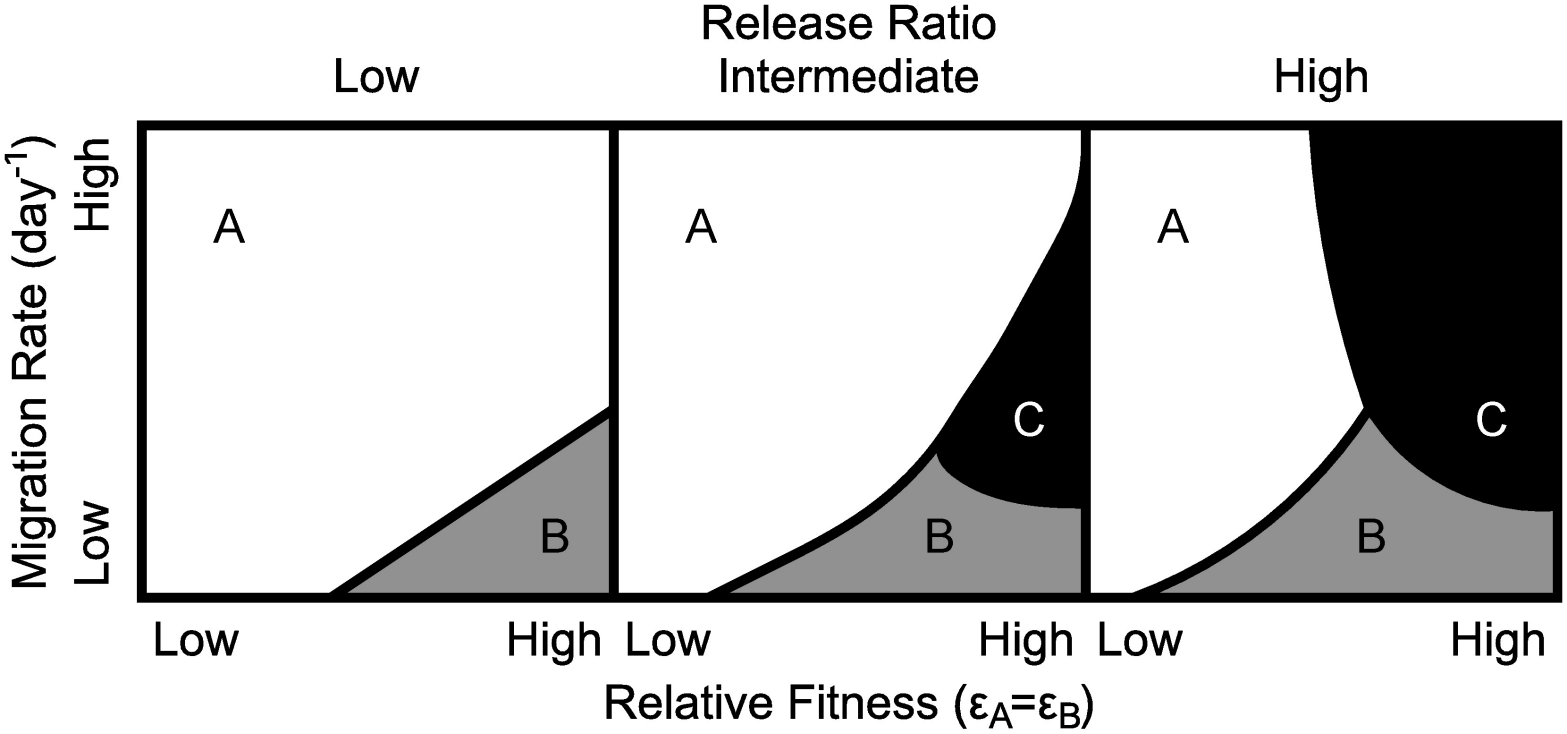
A cartoon showing the general pattern of possible outcomes from numerical simulations of a two-locus engineered underdominance system with migration between a target and a non-target population. Here the three panels represent examples of the outcome of a system for varying release ratios (increasing from left to right). In the white region (A) the system does not drive in either population and transgenes are eliminated. The grey region (B) produces an intermediate equilibrium state in which transgenes achieve some degree of partial introgression into both populations. In the black region (C) the system is efficient enough to drive in both populations. As different system configurations require different parameter values to achieve each possible outcome, axes here do not display actual values. Results from numerical simulations for a single example release ratio under each different genetic system and release scenario are given in S5–S8 Figs. Note that systems with weakly suppressed lethals (e.g S6 & S8 Figs) require larger release ratios in order to produce the same pattern of outcomes as a system with strongly suppressed lethals.

We identify three distinct outcomes for different parameter combinations in the UD system (summarised in Fig 8). Examples of these results obtained using the mathematical model presented here are given in S5–S8 Figs. These are given for each of the sixteen possible combinations of early or late-acting fitness/lethal effects, bisex or male-only release, bisex or female-specific lethals and strong or weak suppression of lethal genes. Additionally, S9 Fig gives results of typical numerical simulations demonstrating the composition of the final equilibria for each outcome.

The first possible outcome from Fig 8 (white area) is the undesirable scenario whereby the system cannot produce lasting transgene introgression in either population. This may occur because the introduced UD system fails to drive in the target population due to either high fitness costs or an insufficient release ratio. Another reason for this possibility is that the system could theoretically drive in the target population if isolated but may be prevented from doing so by an inward flow of wild-type individuals from the non-target population. This may be thought of as migration from the non-target population acting to lower the transgene frequency below the necessary threshold for lasting transgene introgression.

A second possibility (black regions in Fig 8) is that the UD system achieves lasting and near-fixation transgene introgression in both the target and non-target populations. This may occur where transgenes confer only a small fitness cost to individuals carrying them. The rate of migration must then be sufficiently high to exceed the threshold transgene frequency in the neighbouring population yet not so high as to reduce the transgene frequency below the threshold before the system has a chance to establish itself in the target population. This is a potentially undesirable scenario since it would result in the spread of transgenes into non-target areas.

Finally, it is possible that relatively small fitness costs and rates of migration (grey areas in Fig 8) may lead to an intermediate equilibrium in both the target and non-target populations. This is likely to be the desired outcome for the UD system since it produces stable, high frequency transgene presence in the target population with low transgene frequencies in the non-target population. With any degree of migration at all then, in this regime, it will not be possible to achieve complete eradication of wild-types from the target population nor a zero transgene frequency in the non-target population since the two populations constantly exchange individuals.

The final equilibrium attained in each of the systems considered here cannot be higher than the original equilibrium target or non-target population sizes. This is the case for both the overall population and that of wild-type females (as seen in S9 Fig). Thus we would not expect to observe any equilibrium increase in either the prevalence of a virus or the rate of nuisance biting within either population. However, there will be an initial rise in the total population size equal to the introduced transgenic mosquitoes.

A direct comparison with the results of [16] and [17] is not straightforward. Specifically, here we study migration as a rate per day whereas previous models consider rates per generation. Additionally, [16] and [17] consider fitness costs to be additive whereas we assume they are multiplicative. It is unclear exactly how these differences will affect results, however S5–S9 Figs appear to find the same general pattern of outcomes as [16]. There are however a few significant differences between this work and previous studies. Specifically, our results demonstrate how each outcome would be expected to impact on both the target and non-target population sizes. This work also demonstrates that a range of different release scenarios and genetic systems not previously considered (S5–S8 Figs) would be expected to produce broadly the same pattern of outcomes although thresholds will vary case by case.

Comparing these results to those obtained using population dynamics models shows very similar results. There are, however, very small regions around threshold boundaries in which population suppression could prevent UD systems from spreading into a non-target neighbouring population. This is due to the fact that population suppression acts to lower the rate of transgenic migration out of the target population.

**Fig 9:**
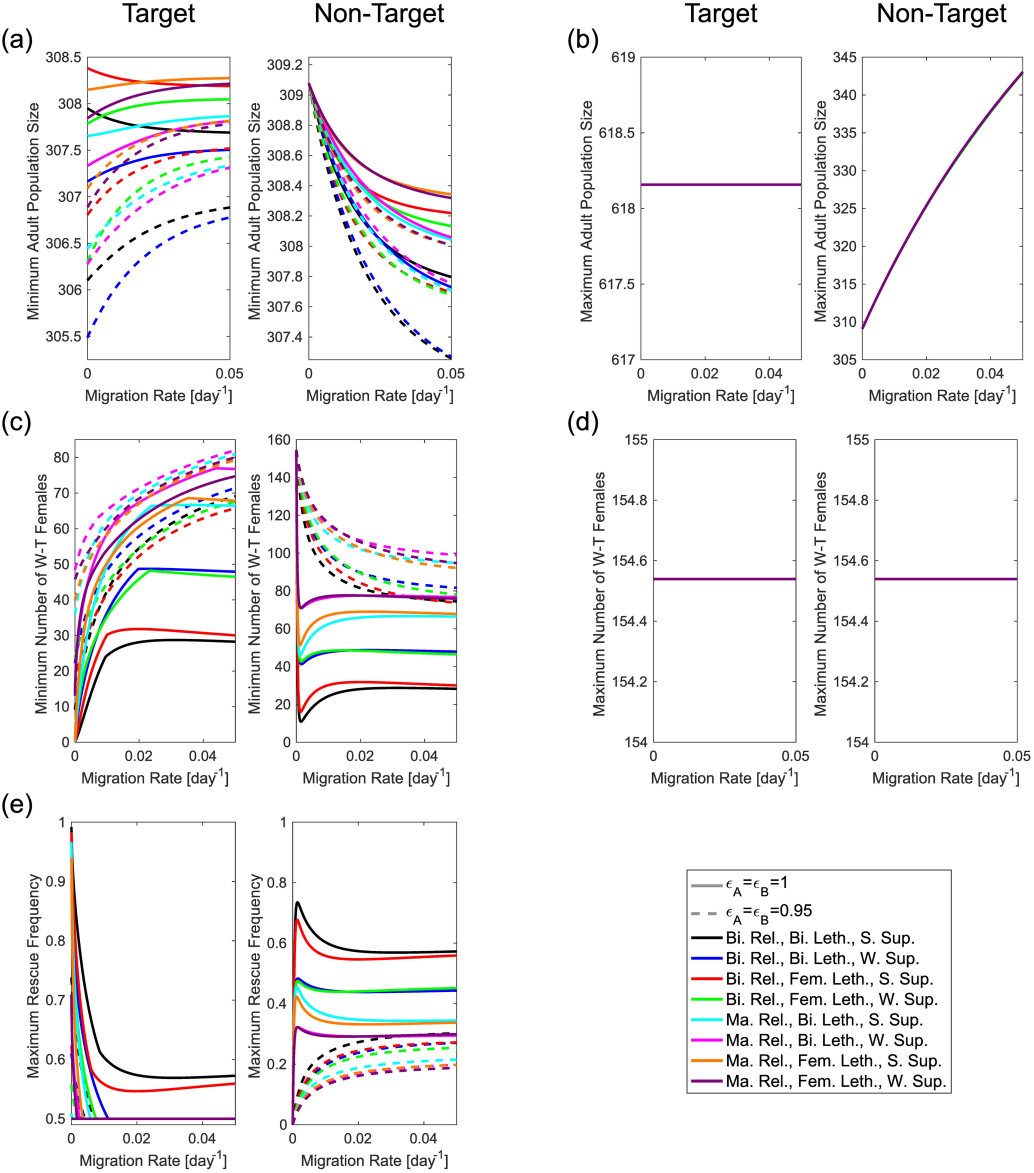
Rates of migration can play a significant role in determining the effects of a killer-rescue system. Results shown here are for a KR system with late-acting fitness/lethal effects introduced into a population of ∼309 individuals (half of which are females). Panels (a) and (b) show the effects of migration rate on the maximum and minimum population sizes (total of all genotypes) attained following the release of a KR system, respectively. Panels (c) and (d) show equivalent results for the maximum and minimum wild-type female population sizes, respectively. Finally, panel (e) shows the effects of migration rate on the maximum rescue transgene frequency that may be attained. In each panel the left-hand plot represents effects on the target population while right-hand plots are for a nearby non-target neighbouring population. In each case, solid lines show results for a KR system that confers zero fitness cost while dashed lines are for a system with 5% fitness cost per construct (i.e. *ϵ*_*A*_ = 1 = *ϵ*_*B*_ and *ϵ*_*A*_ = 0.95 = *ϵ*_*B*_, respectively). Line colours represent a specific configuration of release strategy and genetic system and are detailed in the legend (with Bi. = bisex, Ma. = male-only, Fem. = female-specific, S. = strongly, W. = weakly, Rel. = release, Leth. = lethality and Sup. = suppression of lethals). Panels (b) and (d) appear to display just one line since all results precisely coincide with one another.

Unsurprisingly, results obtained for the KR system (Fig 9 and S10 Fig) under a 1:1 release ratio display a different pattern from those of the UD system. This is likely due to both the differing natures of the two systems and the measures used to assess them. However, as can be seen in Fig 7(c) and 7(d), the KR system does still display some threshold behaviour in that certain conditions on the release ratio and fitness costs of transgenic constructs must be met before the frequency of the rescue construct will increase (i.e. before the KR system will drive).

In particular, Fig 9 and S10(b) and S10(d) Fig show that whilst the maximum size of each population increases from introducing transgenic individuals, there is no increase in the number of adult wild-type female mosquitoes in either population. This holds for all genetic systems and release scenarios considered here, meaning that the introduction of a killer-rescue system would not be expected to result in an increased disease burden since only wild-type females are able to transmit pathogens (assuming cargo genes are fully effective in a single copy).

Fig 9 and S10(a) and S10(c) Fig show that following the transient population increase, we observe some degree of transient suppression of both total and wild-type female population sizes due to the fitness costs and lethality of these systems. In particular, from (a) panels we see that bisex releases produce greater overall population suppression than male-only releases. We also see that bisex lethality produces a greater degree of overall population suppression than female-specific lethality. The strength of lethal suppression doesn’t produce significant differences. For wild-type female populations ((c) panels) we find that bisex releases also produce the greatest degree of population suppression. However, unlike overall population suppression, here we see that strong suppression of lethals produces a much greater effect on wild-type female population sizes than does weak suppression of lethals while bisex or female-specific lethality makes little difference.

In (e) panels of Fig 9 and S10 Fig we see the effects of migration on the maximum transgene frequency attained. In the target population, we see that only bisex releases of genetic systems with strongly suppressed lethals are capable of increasing in frequency for all rates of migration considered here. All other systems considered have a threshold migration rate above which they will not increase in frequency. This threshold migration rate is highest for cases with bisex releases. For male-only releases we find that strong suppression of lethals gives a higher migration threshold than does weak lethal suppression. For the non-target population we see that bisex releases produce greater transgene frequencies than male-only releases. Strong suppression of lethals also produces higher transgene frequencies than weak lethal suppression. For both the target and non-target populations, we observe little difference as a result of bisex versus female-specific lethality.

Each of the results discussed above differ significantly from the case with no release of transgenic mosquitoes. Here the maximum and minimum total adult population sizes will be equal for both populations. The same will be true of the wild-type female populations and these will be exactly half of the total population. Assuming that the two populations are of equal size then the rate of migration will also have no impact on the size of either population.

## Discussion

Within this study we have formulated and analysed a flexible population dynamics model of UD and KR classes of gene drive. This produces results in agreement with the previous literature in terms of necessary introduction thresholds. This work investigated the effects of a number of important ecological factors on the equilibrium size of a mosquito population. Results presented here suggest that, all other things being equal, neither the size of a mosquito population nor the strength of density dependent larval competition will have a major bearing on the final outcome of UD or KR gene drives (assuming the release size is adjusted accordingly), however the density dependence significantly alters the dynamics observed in reaching that outcome. The model was then extended to consider the effects of migration between target and non-target mosquito populations, revealing three distinct outcomes for the UD system. Transgenes may either achieve lasting (near-fixation) introgression into both populations; be eliminated from both populations; or reach an intermediate equilibrium in each. Any of these outcomes may be viewed as desirable in certain circumstances; however the spatially limited drive achieved by the intermediate equilibrium scenario (high and low frequencies in the target and non-target populations, respectively) is traditionally viewed as success for the UD system. For a KR system the effects of migration are not so easily categorised, instead having a more continuous effect. Specifically, increased rates of migration lower the maximum transgene frequency in the target population and raise it in the non-target population. In combination these results suggest that the final outcomes of UD and KR systems are robust to a number of ecological factors and thus represent feasible strategies for replacement of mosquito populations and testing of cargo genes, respectively.

Previously, discrete generation population genetics models have been used to predict the key features/behaviours of UD and KR systems. Some ecological factors have been investigated for the systems studied here. In particular, for UD systems Huang et al. [19, 20] considered models with age and spatial structure. These studies consider a similar form of density dependence to that used here but their model structure considers the age (in days) of larvae and also allows competition between larvae of all ages. For the KR system, a great number of factors are incorporated in the highly complex Skeeter Buster [22] simulation model, however this does not easily allow the effects of each and their interactions to be disentangled. Here, the use of a simpler population dynamics model allows the effects of each ecological parameter to be more readily analysed. This work also examined the effects of differing release strategies (release ratios and sexes released) and genetic systems (sexes affected by lethals and strength of lethal suppression), previously considered for the UD system [18] but not for the KR system.

The models upon which this study is based have been successfully used in the study of alternative genetic control strategies (e.g. the sterile insect technique and mosquito control with homing endonuclease genes) and other pathogens (e.g. malaria) [23, 24]. Thus we expect the model given here may be adapted to represent other species, classes of gene drive, pathogens (e.g. lymphatic filariasis and Zika) and further ecological factors.

As with any modelling study, this work is based upon a number of simplifying assumptions, each of which is common and have been discussed in previous works of a similar type [8, 15, 18, 23, 24]. Thus we do not discuss them any further here. There are however a number of areas in which future work may help to refine our understanding of gene drive efficacy.

Here we consider a fixed time delay as a simple form of age structuring representing the developmental time between egg laying and emergence as adults. As a consequence of studying models such as this, it is implicitly assumed that density dependent competition occurs only between larvae born at the same time. Thus, future work could look to incorporate formalised age structuring (e.g. that of Huang et al. [20]) into a model similar to that studied here. This may also allow us to more precisely evaluate the effects of different timings of transgene fitness costs/lethality. It would also be interesting to compare results given here to those from a model such as that of Huang et al. [20] to see whether the oscillatory behaviour seen in Fig 6 is still feasible.

The models investigated here implicitly assume that adult individuals mate each day and do not store sperm across days. This is a limitation of applying this type of model to mosquito populations. Future work may therefore seek to explore the effects of including more realistic assumptions on the mating behaviours of adult mosquito populations.

The two-deme model formulation used here represents two mosquito populations as each panmictic with no internal spatial structure; essentially as entities existing at single points in space. Future extensions could thus seek to incorporate spatially structured mosquito populations to explore how mosquito behaviour could affect gene drive efficacy.

Throughout this study we assume the absence of mutations and resistance. At present, the probability of these factors occurring is unclear and may only be characterised through long term experiments. However, the techniques of Marshall [50] would allow the effects of transgene separation to be explored theoretically.

We assume that the introduction of UD or KR gene drives associated with a refractory “cargo” gene at a sufficiently high allele frequency in a vector population would reduce disease transmission. An extension of this model that considers human populations and epidemiology would allow this effect to be quantified.

In this work we assumed that the size of each mosquito population is known, such that releases can be made at a predetermined ratio to that number. In practice, the accurate measurement of wild mosquito population sizes is a major technical challenge and one that must be overcome in order to ensure that any releases of UD or KR carrying mosquitoes will have their desired effect.

A key feature of UD and KR gene drives is that they are not predicted to eliminate targeted populations and can therefore potentially disrupt pathogen transmission without leaving a vacant ecological niche into which another species may spread. However, results presented here and in the previous literature have shown that various classes of gene drive can produce a transient phase in which a targeted population may be heavily suppressed (e.g. UD, KR and *Wolbachia* [51, 52]). Since *Ae. aegypti* and *Aedes albopictus* are known to compete [53–55] it is possible that the successful implementation of UD and KR gene drives could lead an existing *Ae. aegypti* population to be displaced by *Ae. albopictus* where it would not otherwise have been. This would likely hamper efforts to eliminate viruses such as dengue since *Ae. albopictus* are also competent vectors [56]. Such competition has been studied in the context of SIT/RIDL control [57] but has not yet been extended to consider classes of gene drive.

This work has demonstrated the feasibility of mosquito control using either UD or KR gene drive systems. In addition to further refining the relationship between specific designs, release strategies and fitness regimes in terms of the constraints they impose on the thresholds for desired outcomes to emerge, this work also helps to elucidate the impacts of a number of ecological factors. Whilst this study should be useful for scientists and regulators when examining/designing UD and KR gene drive systems, further modelling and experimental work would likely help to further refine our understanding of such systems and how they may help in the control of mosquito-borne diseases.

## Supporting information

S1 Appendix Further mathematical modelling details. Section 1 contains further information on the combined population genetics and population dynamics model including initial conditions and transgene frequency calculations for each system. Section 2 provides evidence for the small discrepancies in the results of Figs 6 and 7 being caused by numerical error.
(PDF)

S1 Table Matings between different genotype pairs produce various outcomes in terms of the genotypes of their progeny. The two left-hand columns indicate a male and female mating pair with given genotypes whilst the resultant fractions of their progeny that are of each genotype are outlined in the nine remaining columns.
(XLSX)

S1 Fig Small deviations from the example simulation lines in Figs 6 and 7 are likely caused by numerical error. Each panel here displays lines representing the differences in the maximal degree of population suppression between the eight data sets from Figs 6 and 7. Coloured lines represent the differences between numerical simulations conducted using three different ‘MaxStep’ values within MATLAB (The MathWorks Inc., Natick, MA) delay differential equation solver dde23. Pink lines show differences between simulations with MaxStep = 0.1 and MaxStep = 0.025. Orange lines are differences between simulations with MaxStep = 0.1 and MaxStep = 0.05. Green lines show differences between simulations conducted with MaxStep = 0.05 and MaxStep = 0.025. Simulations conducted using the same MaxStep value produce no differences.
(EPS)

S2 Fig Small variation between results is likely due to the use of different ‘MaxStep’ parameters in MATLAB solver dde23. Shown here are sample numerical simulations of the total number of adult mosquitoes during a time period following the release of a killer-rescue system. The examples shown here are for a 1:1 release of a system with late-acting transgenes and relative fitness per construct of *ϵ*_*A*_ = 0.95 = *ϵ*_*B*_. Coloured lines represent simulations carried out using different ‘MaxStep’ (Δ*t*_*max*_) parameters with black as an example simulation with Δ*t*_*max*_ = 0.005 and other colors representing the parameters used within the main text, i.e. Δ*t*_*max*_ = 0.1 (blue), Δ*t*_*max*_ = 0.05 (red) and Δ*t*_*max*_ = 0.025 (green). Clearly, the use of different ‘MaxStep’ parameters affects how close the minimum of a numerical simulation can be to the true minimum value.
(EPS)

S3 FigInitial population size does not impact upon the dynamics or outcome of two-locus engineered underdominance or killer-rescue gene drive systems.Results here show the percentage change in total mosquito population size (all genotypes) following a 1:1 (introduced:wild) bisex release of transgenic individuals. This is shown for the full range of relative fitness parameters (i.e. 0 ≤ *ϵ*_*A*_ = *ϵ*_*B*_ ≤ 1) over the first 500 days following a release. Note that the 100% increase in population size in each example is due to the initial release of transgenic mosquitoes. Panels in the left hand column are examples created with *α* = 0.02 (corresponding to a population of 309.08) whereas those in the right column are for *α* = 0.7 (i.e. a population of 8.83). The central column shows the difference between the two numerical simulations. Note that the maximum difference between each pair of simulations is of the order $O(10^{-13})$ which is well within the bounds of numerical error and unlikely to represent any mechanistic difference.(EPS)

S4 Fig Strength of density dependence does not significantly alter the evolution of transgene frequencies. Here overall transgene frequencies (for engineered underdominance, panel (a)) and rescue transgene frequencies (for killer-rescue, panel (b)) are shown under three different strengths of density dependence. In particular, simulations are presented for *β* = 1.1 (blue line), *β* = 2.75 (red dashed line) and *β* = 3.5 (green dotted line). The left-hand column shows results over a full numerical simulation whilst the right-hand column shows enlarged versions of the time period *t* = 0−200 days (in the black rectangles) to show extra detail. Note here that transgene frequencies appear to diverge and then begin to converge as the systems reach their final equilibrium.
(EPS)

S5 Fig Migration between neighbouring populations can lead to three possible outcomes from a two-locus engineered underdominance system. Here results are shown for numerical simulations of the two deme population dynamics model presented in the main text. Plots here show the effect, at equilibrium, of a 1:1 (introduced:wild) release of the relevant systems. Each row represents a different release scenario or genetic system, as detailed in the respective titles. Within each row the left-hand pair of plots shows the impact on the overall population sizes of the target and non-target populations whilst the right-hand panels show the effect on just the population of wild-type females (assumed to be the only mosquitoes capable of infecting humans).
(EPS)

S6 Fig Migration between neighbouring populations can lead to three possible outcomes from a two-locus engineered underdominance system. Here results are shown for numerical simulations of the two deme population dynamics model presented in the main text. Plots here show the effect, at equilibrium, of a 10:1 (introduced:wild) release of the relevant systems. Note that a larger release ratio is considered for systems with weakly suppressed lethals due to differences in the introduction thresholds of various systems [18]. Each row represents a different release scenario or genetic system, as detailed in the respective titles. Within each row the left-hand pair of plots shows the impact on the overall population sizes of the target and non-target populations whilst the right-hand panels show the effect on just the population of wild-type females (assumed to be the only mosquitoes capable of infecting humans). It is worth noting here that the male-only release of systems with weakly suppressed lethals does not appear able to produce the region in which lasting introgression occurs within both populations.
(EPS)

S7 Fig Migration between neighbouring populations can lead to three possible outcomes from a two-locus engineered underdominance system. Here results are shown for numerical simulations of the two deme population dynamics model presented in the main text. Plots here show the effect, at equilibrium, of a 1:1 (introduced:wild) release of the relevant systems. Each row represents a different release scenario or genetic system, as detailed in the respective titles. Within each row the left-hand pair of plots shows the impact on the overall population sizes of the target and non-target populations whilst the right-hand panels show the effect on just the population of wild-type females (assumed to be the only mosquitoes capable of infecting humans).
(EPS)

S8 Fig Migration between neighbouring populations can lead to three possible outcomes from a two-locus engineered underdominance system. Here results are shown for numerical simulations of the two deme population dynamics model presented in the main text. Plots here show the effect, at equilibrium, of a 10:1 (introduced:wild) release of the relevant systems. Note that a larger release ratio is considered for systems with weakly suppressed lethals due to differences in the introduction thresholds of various systems [18]. Each row represents a different release scenario or genetic system, as detailed in the respective titles. Within each row the left-hand pair of plots shows the impact on the overall population sizes of the target and non-target populations whilst the right-hand panels show the effect on just the population of wild-type females (assumed to be the only mosquitoes capable of infecting humans). It is worth noting here that the male-only release of systems with weakly suppressed lethals does not appear able to produce the region in which lasting introgression occurs within both populations.
(EPS)

S9 Fig Examples of the potential equilibria obtained from the release of a two-locus engineered underdominance system with migration between a target and a non-target population. Here the three panels represent different example equilibria, namely (a) transgenes achieve lasting introgression into both populations; (b) transgenes achieve lasting introgression into the target population and sustain partial introgression into the non-target population and (c) lasting transgene introgression is achieved in the target population without any significant introgression into the non-target population. Different line colours and styles are used to represent the numbers of mosquitoes for each individual genotype, with details given in the respective legends.
(EPS)

S10 Fig Rates of migration can play a significant role in determining the effects of a killer-rescue system with late-acting fitness/lethal effects. Panels (a) and (b) show effects of migration on the maximum and minimum population size (all genotypes) following the release of a killer-rescue system. Panels (c) and (d) show similar results but for the size of the wild-type female population. Finally, panel (e) shows the effects of migration on the maximum rescue frequency achieved. In each panel the left hand plot represents the target population while the right hand panel is for a non-target neighbouring population. In each case solid lines show results for a system with no fitness costs while dashed lines are for a system with 5% fitness costs per construct (i.e. *ϵ*_*A*_ = 1 = *ϵ*_*B*_ and *ϵ*_*A*_ = 0.95 = *ϵ*_*B*_, respectively). Line colours represent specific combinations of release strategies and genetic systems, as detailed in the legend (with Bi. = bisex, Ma. = male-only, Fem. = female-specific, S. = strongly, W. = weakly, Rel. = release, Leth. = lethality and Sup. = suppression of lethals).
(EPS)

S11 Fig Threshold lines of Fig 7 viewed under a high degree of magnification. Here we can see small differences, with increasing strength of density dependence very slightly raising tolerable fitness costs for the UD system (top row) and very slightly decreasing them for the KR system (bottom row).
(EPS)
