# Supplementary material for: Population dynamics of engineered underdominance and killer-rescue gene drives in the control of disease vectors
Source: PLoS Comput Biol. 2018 Mar 23;14(3):e1006059. doi: 10.1371/journal.pcbi.1006059 (PMC5884568; doi:10.1371/journal.pcbi.1006059)
Supplement: S1 Appendix — Section 1 contains further information on the combined population genetics and population dynamics model including initial conditions and transgene frequency calculations for each system. Section 2 provides evidence for the small discrepancies in the results of Figs 6 and 7 being caused by numerical error. (PDF) [file pcbi.1006059.s001.pdf]

Supplementary Information: Population dynamics of engineered underdominance and killer-rescue gene drives in the control of dengue vectors

Matthew P. Edgington and Luke S. Alphey

The Pirbright Institute, Ash Road, Woking, Surrey GU24 0NF, UK

1 Modelling

Within the main text we outline the key details of the mathematical models used throughout this work. Here we present some extra details necessary to reproduce the results of this study. We begin with the $v_i(t-\tau)$ and $w_i(t-\tau)$ expressions that are omitted from the main text for brevity. These expressions represent the outcomes from each possible genotype mating pair and are grouped according to the genotypes of their resulting progeny. For example, the expression used to represent all genotype mating pairs resulting in wild-type progeny is of the form

$$\begin{aligned} v_1(t-\tau) = \frac{1}{N(t-\tau)} \Bigg[& M_1(t-\tau)F_1(t-\tau) + \frac{1}{2}M_1(t-\tau)F_2(t-\tau) + \frac{1}{2}M_1(t-\tau)F_4(t-\tau) \\ & + \frac{1}{4}M_1(t-\tau)F_5(t-\tau) + \frac{1}{2}M_2(t-\tau)F_1(t-\tau) + \frac{1}{4}M_2(t-\tau)F_2(t-\tau) + \frac{1}{4}M_2(t-\tau)F_4(t-\tau) \\ & + \frac{1}{8}M_2(t-\tau)F_5(t-\tau) + \frac{1}{2}M_4(t-\tau)F_1(t-\tau) + \frac{1}{4}M_4(t-\tau)F_2(t-\tau) + \frac{1}{4}M_4(t-\tau)F_4(t-\tau) \\ & + \frac{1}{8}M_4(t-\tau)F_5(t-\tau) + \frac{1}{4}M_5(t-\tau)F_1(t-\tau) + \frac{1}{8}M_5(t-\tau)F_2(t-\tau) + \frac{1}{8}M_5(t-\tau)F_4(t-\tau) \\ & + \frac{1}{16}M_5(t-\tau)F_5(t-\tau) \Bigg], \end{aligned} \tag{S1}$$

where N denotes the total number of mosquitoes within a population; M_i and F_i (where $i = 1, ..., 9$) represent the number of male and female mosquitoes of genotype i; t represents the current time within a simulation; and τ represents the egg to adult developmental delay time. The remaining v_i expressions are of the same form as Eq (S1) with the contents of the square brackets representing the entries in the i-th genotype column in S1 Table. Note that the expressions for w_i are the same as those for v_i but with $M_i(t-\tau)$ variables swapped for $F_i(t-\tau)$ and vice versa.

A number of times within the main text we refer to the consideration of given release ratios

- usually 1:1 (introduced:wild). This represents the ratio of introduced mosquitoes (only release of genotype $AABB$ (i.e. $i = 9$) considered within this work) to the wild population at the time of the release. In order to implement this within the mathematical model we assume that the wild population size is in equilibrium at the time of release (as defined by Eq (14) of the main text). In some situations it may be necessary to consider alternative release ratios, for example the 10:1 (introduced:wild) release ratio considered in S6 Fig and S8 Fig. This may be achieved by considering a set of initial conditions of the form

$$M_1(0) = \frac{N^*}{2}, \quad M_{2-8}(0) = 0, \quad M_9(0) = \theta\frac{N^*}{2}, \quad F_1(0) = \frac{N^*}{2}, \quad F_{2-8}(0) = 0, \quad F_9(0) = \theta\frac{N^*}{2}, \tag{S2}$$

where θ is the release ratio parameter which sets a release ratio of θ:1 (introduced:wild) and must be chosen such that $\theta \geq 0$ in order to be biologically realistic. We also consider here initial conditions covering the duration of the developmental delay time. These take the form

$$M_1(t) = \frac{N^*}{2}, \quad M_{2-9}(t) = 0, \quad F_1(t) = \frac{N^*}{2}, \quad F_{2-9}(t) = 0, \tag{S3}$$

and apply over the time period $\tau \leq t < 0$.

Finally, in main text Fig 1 and S4 Fig results are presented in terms of relevant genotype frequencies for each system since this can be simpler to visualise and understand than the evolution of each individual genotype. Due to the differences between the engineered underdominance (UD) [1] and killer-rescue (KR) [2] systems considered here it is necessary to consider a different frequency for each. For the UD system both transgenic constructs carry the cargo (refractory) gene and so we consider the overall transgene frequency, calculated as

$$\text{Transgene Freq.} = \frac{\frac{1}{4}\left(G_2 + G_4\right) + \frac{1}{2}\left(G_3 + G_5 + G_7\right) + \frac{3}{4}\left(G_6 + G_8\right) + G_9}{\sum_{i=1}^{9} G_i}. \tag{S4}$$

This differs from the killer-rescue system for which only the rescue transgene contains the cargo (refractory) gene. As such, for this system we are only interested in the frequency of the rescue

transgene which is calculated as

$$\text{Rescue Freq.} = \frac{\frac{1}{2}\left(G_2 + G_5 + G_8\right) + G_3 + G_6 + G_9}{\sum_{i=1}^{9} G_i}. \tag{S5}$$

Note that within these expressions numbers of males and females of each genotype have been summed according to $G_i(t) = M_i(t) + F_i(t)$.

2 Variation in results is likely caused by numerical error

Figs 6 and 7 of the main text display results of numerical simulations created for the UD and KR systems over the whole range of possible relative fitness parameters ($0 \leq \epsilon_A, \epsilon_B \leq 1$) and a selection of different initial population sizes. As mentioned in the main text, upon close inspection it can be seen that there are small differences between the results from the individual data sets and the randomly selected example data set. Further examination of these results suggests that the deviations are due to differences in the numerical errors present in each set of numerical results. Plots showing results that support this notion are given in S1 Fig.

These results display a number of characteristics that lead us to conclude that the observed deviations from the randomly chosen example case are caused by numerical error. Firstly, the data collections for each initial population size required that different maximum time steps ('MaxStep') were used as options in MATLAB (The MathWorks Inc., Natick, MA) delay differential equation solver dde23 [3] in order to produce results such as those in S3 Fig. This is because the 'surf' plotting command in MATLAB requires that, to connect results from individual simulations as a surface, they all consist of the same number of time points. To ensure that this was the case for each data set collected we utilise three different 'MaxStep' parameters for both UD and KR simulations which are

- MaxStep=0.1 (for $\alpha = 0.7,\ 0.2,\ 0.07$ and 0.02);
- MaxStep=0.05 (for $\alpha = 0.007$ and 0.002); and
- MaxStep=0.025 (for $\alpha = 0.0007$ and 0.0002).

Since we utilise three different 'MaxStep' parameters during the collection of data sets, it appears likely that these correspond to the three distinct error lines in S1 Fig. Thus, we propose here that the reason for the existence of just three error lines is that there are only differences between cases that utilise different 'MaxStep' parameters. It is also the case that those examples which used smaller 'MaxStep' parameters displayed the largest results for the maximum degree of population suppression in both the UD and KR systems. This is likely due to the fact that a smaller 'MaxStep' means that the simulation would capture a data point closer to the actual minimum whereas the larger 'MaxStep' parameters may result in simulations with consecutive time steps lying further from the absolute minimum. S2 Fig shows an example of how such a numerical error could be produced through the use of differing 'MaxStep' values.

Another feature that suggests these differences are due to numerical error is the very small absolute size of deviations from the randomly chosen example simulation and between data sets. In particular, S1 Fig shows maximal absolute errors between data sets of $\sim 1.3 \times 10^{-5}$ and $\sim 2.25 \times 10^{-4}$ for the cases with early- and late-acting fitness/lethal effects, respectively. In addition to the fact that the sizes of variations between data sets are consistent with what we would expect from Matlab solver dde23, we chose an optional relative error tolerance ('RelTol') parameter equal to 10^{-5}. The use of such a relative error tolerance parameter could also explain why we see greater variation between the data sets in cases with late-acting rather than early-acting fitness/lethal effects (i.e. since the late-acting examples display larger absolute changes over a single time step, the relative error tolerance would allow for larger errors in results and thus between data sets).

References

[1] S. Davis, N. Bax, and P. Grewe. Engineered underdominance allows efficient and economical introgression of traits into pest populations. *Journal of Theoretical Biology*, 212(1):83–98, 2001.

[2] F. Gould, Y. Huang, M. Legros, and A.L. Lloyd. A Killer–Rescue system for self-limiting gene drive of anti-pathogen constructs. *Proceedings of the Royal Society of London B: Biological Sciences*, 275(1653):2823–2829, 2008.

[3] L.F. Shampine and S. Thompson. Solving DDEs in MATLAB. *Applied Numerical Mathematics*, 37(4):441–458, 2001.
